# Cytokines and Chemokines as Mediators of Prostate Cancer Metastasis

**DOI:** 10.3390/ijms21124449

**Published:** 2020-06-23

**Authors:** Timothy O. Adekoya, Ricardo M. Richardson

**Affiliations:** Julius L. Chambers Biomedical/Biotechnology Institute and Department of Biological & Biomedical Sciences, North Carolina Central University, Durham, NC 27707, USA; mrrichardson@nccu.edu

**Keywords:** prostate cancer, metastasis, cytokines, chemokines

## Abstract

The consequences of prostate cancer metastasis remain severe, with huge impact on the mortality and overall quality of life of affected patients. Despite the convoluted interplay and cross talk between various cell types and secreted factors in the metastatic process, cytokine and chemokines, along with their receptors and signaling axis, constitute important factors that help drive the sequence of events that lead to metastasis of prostate cancer. These proteins are involved in extracellular matrix remodeling, epithelial-mesenchymal-transition, angiogenesis, tumor invasion, premetastatic niche creation, extravasation, re-establishment of tumor cells in secondary organs as well as the remodeling of the metastatic tumor microenvironment. This review presents an overview of the main cytokines/chemokines, including IL-6, CXCL12, TGFβ, CXCL8, VEGF, RANKL, CCL2, CX3CL1, IL-1, IL-7, CXCL1, and CXCL16, that exert modulatory roles in prostate cancer metastasis. We also provide extensive description of their aberrant expression patterns in both advanced disease states and metastatic sites, as well as their functional involvement in the various stages of the prostate cancer metastatic process.

## 1. Introduction

Prostate cancer is the most diagnosed nonskin cancer type in men and remains a major cause of cancer-related deaths among the male population. It is a complex disease that exhibits molecular, pathological, and genomic heterogeneity. Prostate tumorigenesis is a multi-stage process that begins with the development of a low-grade prostatic intraepithelial neoplasia (PINs), which transits into an aggressive adenocarcinoma, then castration-resistant prostate cancer (CRPC), and ultimately advances to become metastatic prostate cancer [1,2]. Because normal prostate tissues rely on androgen and its receptor, androgen receptor (AR), for development and maintenance of homeostasis, targeting the AR pathway via androgen deprivation therapy (ADT) constituted a viable mechanism that was generally utilized for treatment of prostate cancer. Although surgery and radiation are also effective therapy options for localized prostate cancer, ADT remains the first treatment option in metastatic prostate cancer [3,4]. The involvement of AR in modulation of differential gene transcription programming in both AR-dependent and AR-independent prostate cancer has also been reported [5]. ADT resistance ultimately leads either to the development of a primary CRPC or a metastatic CRPC [6]. New guidelines in recent years, however, includes combining ADT with other chemotherapeutic drugs (e.g., Docetaxel) to improve overall patient survival [7,8]. Furthermore, various studies have shown how androgen-dependent and -independent pathways promote prostate tumorigenesis [2,9,10,11,12,13]. In spite of the successes attained in treatment of prostate cancer, these achievement milestones have been dampened by resistance to drug treatments and generation of evasive mechanisms by tumor cells. As a consequence, this disease remains a major healthcare challenge to date.

Most deaths from prostate cancer are as a result of the development of a metastatic disease state [6]. With tumor spread, patients succumb to the terminal stage of prostate tumorigenesis. Prognosis and treatment options at this stage of the disease are low. Metastatic prostate cancer patients were predicted in 98% of cases to have an overall survival of less than 5 years [14]. Prostate tumor cells have the bone as their major site of metastasis and typically appear as osteoblastic lesions interspersed with osteolytic areas [15]. Other organs of metastasis include the lymph node, liver, lungs, and brain [16,17,18]. In general, metastatic prostate cancer is grouped under two main categories: ADT-naïve and ADT-resistant prostate cancer [7]. Other known prostate cancer phenotypes include neuroendocrine (NE) and small cell prostate cancer that are characterized as AR negative and appear as highly aggressive disease forms. These tumor types exhibit aberrant gene mutations and expression, which although mainly impacts AR, may also involve other genes including TP53, PTEN, RB1, ETS, and SPOP among others [7,19]. Taichman et al. [20] described how the generation and maintenance of bone metastatic microenvironment involves a complex interplay of divergent factors that includes bone cells, tumor cells, endothelial cells, immune cells, cytokines and chemokines, as well as an array of growth factors. With metastasis, only a few migrated tumor cells are able to re-establish clones and form macrometastases in the new microenvironment; others loose viability in the blood stream, fail to initiate growth after extravasation, or the generated micrometastases are unable to proceed with their development [21,22].

## 2. Cytokines and Chemokines

Cytokines are a diverse family of low-molecular weight proteins involved in the mediation of communication between cells. They exhibit complex roles in immunity, host defense, inflammation, as well as in tumor immunobiology by acting via autocrine, paracrine, and/or endocrine mechanisms. The major subgroups of cytokines includes interleukins, interferons, colony-stimulating factors, chemokines, as well as tumor necrosis factors, and they are produced either as secreted or membrane-bound proteins [23,24]. A characteristic feature of cytokines is pleiotropy and redundancy; with different cytokines exhibiting functional similarities [25,26,27]. Cytokines elicit their effects by interacting with members of a family of cytokine receptors that includes type I, type II, immunoglobulin superfamily, TNF, G-protein coupled (chemokine), TGFβ, and IL-17 receptors [28]. Upon binding to receptors on target cells, cytokines activate a sequence of downstream proteins that culminates in alteration of gene expression patterns and elicitation of desired responses in target cells [29]. Of these, the Janus kinases (JAK)—signal transducers and activators (STAT) pathway constitutes the canonical pathway activated following cytokine–receptor interaction [30,31,32,33]. Other activated signaling pathways include the PI3K/AKT and Raf/MEK/ERK pathways [33,34,35].

Chemokines are a large family of chemotactic cytokines that modulate immune cell movement and positioning and act by coupling to seven-transmembrane protein receptors known as G-protein coupled receptors (GPCRs). In humans, they are about 50 known chemokines and 20 GPCRs [36,37]. Based on the first two N-terminal cysteine amino acid residues, chemokines are classified into four subfamilies, namely: CC, CXC, CX3C, and XC [38]. Studies have also reported the existence of these proteins either in monomeric, dimeric, or oligomeric states and their molecular form may impact their biological functions [39,40,41]. By interacting with their receptors, chemokines transduce their responses by activating multiple signaling pathways including the PI3K, MAP kinase, and JAK/STAT pathways [42].

Of key importance in tumor progression and metastasis is the modulatory involvement of cytokines and chemokines. Moreover, tumor cells themselves do express cytokines. Cytokine/chemokines impact tumorigenesis by either directly regulating tumor cell growth, invasiveness, and metastasis or indirectly by exerting modulatory effects on stromal cells, immune cells, promotion of metastatic niche, as well as inducing angiogenesis in the cancer microenvironment [24,43,44]. Interestingly, as cancer progresses, the level of expression of numerous cytokines/chemokines and their receptors have been found to correspondingly increase in primary tumor tissues, metastatic sites, and patient serum, with numerous studies revealing correlation between their upregulated expression and tumor progression, metastasis and disease prognosis [45,46,47,48,49]. Similarly in prostate cancer, the involvement of various cytokines/chemokines and their signaling pathways in promotion of tumor growth and metastasis has been well studied [50,51,52,53]. This has been achieved through the use of in-vitro cell-based analysis approaches, human clinical sample analyses, and in-vivo animal xenograft, orthotopic as well as transgenic model systems. Among the most important cytokines/chemokines associated with prostate cancer metastasis and discussed herein includes IL-6, CXCL12, TGFβ, CXCL8, VEGF, RANKL, CCL2, CX3CL1, IL-1, IL-7, CXCL1 and CXCL16.

## 3. Role of Cytokines and Chemokines in Prostate Cancer Metastasis

Metastasis is a process wherein select subpopulations of tumor cell clones detach and disseminate from primary foci, travel through the blood and/or lymph vasculature, and re-establish themselves in a secondary growth site. Metastasis involves a complex cascade of events that includes primary tumor site extracellular matrix (ECM) remodeling, epithelial–mesenchymal transition (EMT), angiogenesis, basement membrane invasion, intravasation, travel of circulating tumor cells (CTCs) through the blood, extravasation, generation of a premetastatic niche and culminates with homing, and re-establishment of tumor cells in the metastatic site [16,54,55,56]. It is understood that CTCs migrate to the bone marrow using similar mechanisms adopted by hematopoietic stem cells during bone marrow transplantation [57]. The entire metastasis process is regulated by multiple signaling pathways and with disseminating tumor cells undergoing an array of biochemical, molecular and phenotypic changes. During the initial phase of prostate cancer metastasis, tumor cells undergo gene changes that allows for both diminished cell–cell and cell–ECM adhesion as well as increased invasiveness and migratory ability [21]. Substances are secreted into the tumor microenvironment (TME) which causes degradation of the ECM and basement membrane, chemotaxis of immune cells, alteration of adhesion protein expression, and remodeling of the primary tumor site. Some of these secreted substances include proteins known as cytokines and chemokines.

**Figure 1:**
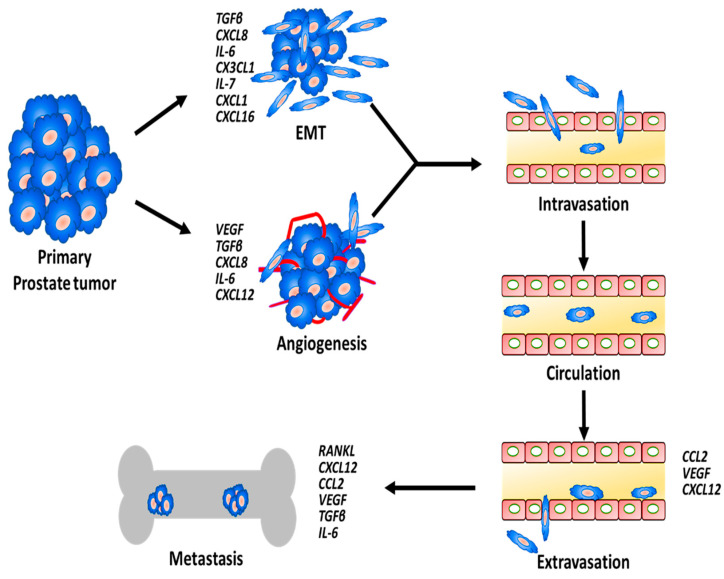
Schematic showing the process of prostate cancer cell metastasis and the involvement of various cytokines and chemokines along the sequence of event.

Cytokines and chemokines secreted by prostate cancer cells, stromal cells, immune cells, and other cells within the TME as well as metastatic site drive the various stages of the metastasis process (Figure 1). These proteins exert their effects either through autocrine or paracrine mechanisms and facilitate cross talk between prostate tumors and the TME.

### 3.1. Cytokines in Prostate Cancer EMT

A characteristic feature of metastatic cells is the ability to undergo EMT. It is a sequence of events that transforms differentiated epithelial cells into undifferentiated mesenchymal phenotype and confers invasive and migratory ability on them [55,58,59]. This transdifferentiation process allows tumor cells to become more mobile, as they generate invasive protrusions and loose cell–cell contacts [55,58]. During EMT, E-cadherin expression is downregulated while N-cadherin and vimentin expression are upregulated, along with increased metalloproteinase (MMP) expression [16,55,60]. Both EMT and its reverse process, mesenchymal–epithelial transition (MET), are both required for metastasis initiation and progression, respectively [61]. Recent studies, however, indicate that tumor cells may actually exist in different phases along the EMT spectrum as they metastasize and not just completely switch to mesenchymal phenotype as previously suggested [62,63]. EMT phenotypic changes have also been implicated in the development of stem-like properties and it constitutes a major driver of drug resistance in cancer cells [59,64,65]. Furthermore in a metastatic prostate cancer, a large proportion of CTCs present with epithelial, mesenchymal, and stem-cell markers co-expression [66]. Consequently, several studies have reported association between alteration in expression of EMT markers and prostate cancer progression and metastasis [67,68,69,70,71,72,73].

Cytokines have found undisputable roles in the process of EMT; among which includes TGFβ, IL-6, CXCL8, IL-7, and CX3CL1 [73,74,75,76,77]. Stimulation of ARCaP cells by TGFβ1, along with EGF, promoted EMT and resulted in increased incidence of bone metastasis [78]. Chen et al. [79] reported the ability of TGFβ to promote EMT by downregulating the expression of human leukocyte antigen class I (HLA-1) in prostate cancer cells. Similarly, the induction of EMT by TGFβ in prostate cancer was found to be mediated through TRPM7 modulation [80]. Giannoni et al. [81] described how prostate cancer-derived IL-6 secretion stimulated release of MMPs from cancer-associated fibroblasts within TME and this resulted in the promotion of tumor growth and angiogenesis, via EMT and upregulation of stem-cell markers in the tumor cells.

### 3.2. Cytokines in Prostate Cancer Angiogenesis

Enhanced angiogenesis is one of the hallmark processes involved in cancer metastasis, prostate cancer inclusive, and has remained a target for prostate cancer treatment [82]. This is because increased neovascularization and oxygenation facilitates tumor growth, invasion, and metastasis. Tumor-associated angiogenesis is driven by various cytokines including vascular endothelial growth factor (VEGF), CXCL8, IL-6, and TGFβ [83,84,85]. As tumor begins to develop, these proteins induce angiogenic switch within the prostate TME by initiating change from a prevascular to a vascularized phenotype, and this is characterized by increased proliferation and migration of endothelial cells as well as increased formation of vascular tubes [86]. During this process, there is a consequential breakdown of the ECM and basement membrane and this promotes tumor cell intravasation [87].

Metastatic prostate cancer cell lines demonstrate increased gene expression of proangiogenic cytokines VEGF, CXCL8, and TGFβ [88]. Among the known proangiogenetic cytokines, VEGF remains the prime and most potent cytokine, exerting high mitogenic actions on endothelial cells [89]. Prostate cancer cell lines as well as primary cultures of human prostate cancer clinical samples all express VEGF [90]. Inhibition of the VEGF/VEGFR axis suppresses prostate tumor angiogenesis and metastasis [91,92,93]. Intratumoral lymphangiogenesis is also impacted by the level of VEGF secretion. As shown by Wong et al. [94] using an orthotopic mouse model, siRNA targeted inhibition in VEGF-C resulted in an overall decrease in the number of lymphatic vessels draining through the tumor. Furthermore in the bone, VEGF facilitates creation of a premetastatic niche and allows tumor cell homing into skeletal tissues [87].

Angiogenic roles of TGFβ have also been reported. Zhang et al. [95] showed how TGFβ modulates prostate tumor growth and angiogenesis via its regulatory actions on CXCL8 expression levels. Blocking TGFβ signaling, by overexpressing a dominant negative TGFβ type II receptor, decreased intratumor vascular staining and prostate cell metastasis [95]. Similarly, tumors treated with TGFβ inhibitors were found to exhibit diminished tumor size, blood vessel formation, and microvesicle density [96].

### 3.3. Cytokines and Homing to Metastatic Sites

CTCs that survive the unfavorable circulation conditions must extravasate and re-establish in the secondary site. For this process to occur, CTCs initially get arrested and adhere to activated endothelial cells before migrating into the metastatic sites, or they may form emboli that rupture blood vessels to penetrate into secondary metastatic sites. It is now known that primary tumors selectively and aggressively modify future metastatic seeding sites, even before CTCs travel occurs [97]. For example, the preference of prostate cancer cells for adhesion to bone marrow endothelium has been suggested as being responsible for the high affinity of prostate cancer metastasis to bone tissues [98,99]. The formation of the premetastatic niche and the establishment of metastasis is driven by the actions of soluble factors and extracellular vesicles released by tumor cells, and these encourage remodeling of distant metastatic sites [97,100]. In prostate cancer, however, little is known about the exact process of formation of the premetastatic niche [97]. Indeed, the cross talk between tumor cells and metastatic microenvironment remains an important element required for promotion of metastasis, with this process involving the activation of multiple signaling pathways and transcriptional processes [101].

Bone tissues constitute the main site of metastasis of prostate tumors. Cytokines such as IL-6, VEGF, CXCL12, CCL2, RANKL, and TGFβ have found essential roles in the creation of premetastatic niche, endothelial attachment of CTCs, promotion of extravasation, remodeling of microenvironment, and establishment of viable macrometastases [102,103]. It is important to note that not all extravasated CTCs survive the new tissue microenvironment. Often times, many undergo a state of dormancy, while others remain as nonviable micrometastases [104,105]. The ability of initially formed micrometastases to progress into macrometastases requires neovascularization of the newly formed metastases; and this is often driven by VEGF secretion, which induces vascularization and nutrient supply [106]. Similarly, VEGFR-1-positive bone marrow progenitors have been reported as being involved in initiation of tumor premetastatic niche formation [107]. Indeed, activation of the VEGF/VEGFR axis is key for establishment of tumor metastasis.

Another important cytokine that promotes CTCs homing is CXCL12, and the enhanced activation of the CXCL12/CXCR4 axis has been linked with prostate cancer metastasis. CXCL12 is a homeostatic chemokine secreted by stromal cells in the bone marrow (including osteoblast) and high expression of CXCL12 is observed in metastatic tissues of prostate cancer [103]. Prostate cancer cells express high levels of CXCR4, which via a concentration gradient migrate by chemotaxis towards the high CXCL12 expressing bone tissues [108,109]. Using a metastatic mouse model, e.g., Shiozawa et al. [110] reported how prostate cancer cells home to bone tissues by targeting the hematopoietic stem cell niche. Furthermore, the decreased secretion of CXCL12 by annexin knockout bone marrow stromal cells was reported as significantly reducing prostate cancer cell migration and binding [111]. CXCL12 may also be involved in arrest of CTCs to endothelial cells as prostate cancer cells activation by CXCL12 promoted upregulation of cell surface adhesion molecules and enhanced bone metastasis [112].

Finally, within the bone metastatic microenvironment, osteoblastogenesis, and bone resorption are key remodeling processes that occur, as prostate tumors establish themselves in the secondary site. Interestingly, IL-6, CXCL12, RANKL, CCL2, and TGFβ secreted by both tumor and bone stromal cells are well-studied cytokines that have been implicated in induction of this process [113,114,115,116]. Festuccia et al. [117] revealed how PC3 cell invasiveness was enhanced following its treatment with osteoblast-derived conditioned media that was found to contain high amounts of TGFβ. In assessing the role of the RANKL/RANK axis in prostate metastasis, it was found that prostate cells release soluble factors that induce increased RANKL expression, proliferation of pre-osteoblast cells, and promoted metastasis [118]. Furthermore, Zhang et al. [119] also established the induction of osteoclastogenesis by prostate cancer cells in a metastatic mouse model by treating animals with the decoy receptor, osteoprotegerin.

In general, the various stages of prostate cancer metastasis that cytokines and chemokines exert functional roles are herein presented in Table 1.

## 4. Cytokines Involved in Prostate Cancer Metastasis

### 4.1. TGFβ

TGFβ is known to possess dual functionality in tumorigenesis: acting both as a tumor suppressor during the earlier stages of cancer and as a tumor promoter in more advanced and metastatic stages [169]. TGFβ has been implicated in various stages of the prostate cancer metastasis process; chiefly in EMT, primary tumor remodeling, angiogenesis, and re-establishment of tumors in the metastatic site [169,170,171]. TGFβ can be secreted either by host immune cells or by prostate cancer cells. TGFβ induces the transformation of the extracellular environment to become prometastatic via a complex interplay of exchanges of tumor cells with both stromal and extracellular matrix [172]. TGFβ binds to its serine-threonine kinase receptors type I and type II, while its signaling is mediated via canonical SMAD- and non-SMAD-dependent pathways. TGFβ promotes EMT by inducing ZEB and SNAIL protein expression, which represses E-cadherin levels while increasing the expression of N-cadherin and vimentin [173,174]. This results in a more enhanced metastatic phenotype.

Tumors and serum of prostate cancer patients have been reported to possess high amounts of TGFβ, which has been found to correlate with a more aggressive and metastatic disease [175]. Enhanced production of TGFβ1 and decreased TGFβ type II receptor expression constitute poor prognosis factors due to raised metastatic and angiogenic potential in prostate cancer [176]. Similarly in bone metastasis, there is enhanced activation of TGFβ signaling [173]. TGFβ promotes cell–cell changes and integrin-ECM remodeling, as well as causes rearrangement of the cytoskeleton structure of tumor cells to facilitate increased motility [177]. The prometastatic effect of this cytokine has been established in numerous studies wherein various downstream mediators of this pathway have been assessed. As reported by Hansen et al. [178], the expression and shedding of the cell adhesion molecules, ALCAM, is increased through TGFβ signaling in metastatic prostate cancer cells. As described in their study, diminishing ALCAM expression in the bone metastatic PC3 cells corresponded to decreased tumor growth and metastasis [178]. Elevated levels of a member of the TGFβ superfamily, Activin A, has also been linked with prostate cancer metastasis [123].

Loss of the TGFβ signaling has also been shown to be an augmenting factor that hastens metastasis of prostate cancer. Using a transgenic SV 40 T-antigen-driven mouse prostate model with a dominant negative TβRII mutant receptor, it was reported that disruption of the TGFβ signaling promoted prostate cancer metastasis to the lymph node, lungs, and liver [179]. The presence of a defective dominant negative TGFβRII receptor in a TRAMP mouse model was found to induce EMT thereby producing a more mesenchyma phenotype and enhanced prostate malignancy [120]. Similarly in a PTEN-null mouse model, genetic depletion of Smad4 resulted in emergence of more invasive and metastatic prostate cancer when compared to tumors from normal PTEN-null animals that possessed enhanced TGFβ/BMP-Smad4 pathway activation [180]. Furthermore using PC3 and DU-145 cells, it was reported that the delivery of TGFβ-targeted oncolytic adenoviruses inhibited bone metastasis in a prostate cancer mouse model [124]. Using PacMetUT1 cells, suppression of TGFβ signaling via shRNA knockdown of TGFβ1 or usage of inhibitors in a metastatic nude mouse model further revealed how TGFβ impacts osteoblastic metastasis of prostate cancer [181]. Interestingly, the antimetastatic actions of various compounds are capable of being reversed by TGFβ-induced EMT and its cross talk with MMP upregulation [121,122].

### 4.2. IL-6

IL-6 is a pleiotropic pro-inflammatory cytokine which has been shown to be involved in prostate tumorigenesis and with actions mediated via autocrine and paracrine mechanisms. It has been found to play roles in EMT, angiogenesis, and bone remodeling. By binding to its receptor, IL-6R, its actions are elicited by multiple pathways, specifically through the JAK/STAT as well as by Ras/MAPK and PI3K signaling pathways [182,183,184]. Several studies have reported IL-6 as a prognostic factor in prostate cancer, with elevated serum levels found in patients with metastatic disease [185,186,187]. In bone metastatic patients for example, levels of both IL-6 and soluble IL-6 receptor (IL-6-SR) has been found to be increased [188]. In fact, IL-6 has been implicated as a prime contributory element responsible for the development of cachexia in prostate cancer patients [189].

In human prostate cancer cells, the role of IL-6 in promotion of metastasis has been extensively described. Using LNCaP, DU-145, and LAPC4 cell lines, Santer et al. [190] described how the process of metastasis in prostate cells is increased following IL-6 trans-signaling. Similarly, the suppression of IL-6 signaling axis in hormone-resistant TRAMP-C1 cells was shown to decrease EMT transition and tumor aggressiveness [125]. Overexpression of IL-6 and initiating its signal induction in DU-145 and CWR22Rv1 cells enhanced prostate metastasis, whereas the pharmacological inhibition of JAK2, using AZD1480, suppressed IL-6-induced STAT3 signaling pathway and diminished end-organ metastasis [126].

IL-6 expression has been implicated as one of the main cytokines involved in creating a favorable niche, via bone remodeling, for re-establishment of tumor cells into the metastatic site. One of the mechanisms by which IL-6 alters the bone metastatic microenvironment is by JAK/STAT-mediated promotion of osteoblastic cell differentiation towards a mature phenotype [128]. Morrissey et al. [191] reported that soluble IL-6R was required for IL-6 inhibition of osteoblast-like MC3T3-E1 cell mineralization and that PC3-conditioned media induced osteoclastogenesis-related factors. PC3-conditioned media, found to contain high amounts of IL-6 and IL8 was reported to induce human blood CD11b+ cells osteoclastic differentiation and bone resorption [129]. Using a bone metastatic mouse model, the suppression of IL-6 expression in human PC-3MM2 cells, along with clodronate liposome treatment resulted in decreased tumor size, osteoclast formation, and lowered lymph node metastasis when compared to control animals [127].

Challenging the present dogma of IL-6 role in promotion of prostate cancer metastasis is a recent study. Using PTEN-deficient prostate cancer mouse model, the authors reported enhanced metastasis due to STAT3 inactivation and showed its alteration of the ARF-Mdm2-p53 axis [192]. Increased occurrence of prostate cancer metastasis was also shown to be present in patients with co-deletion of STAT3 and CDKN2A [192]. IL-6 secreted by endothelial cells within the prostate TME are also capable of promoting tumor invasiveness and metastasis via MMP9 activation by causing tumor remodeling [193].

### 4.3. CCL2

CCL2, also known as monocyte chemoattractant protein 1 (MCP-1), belongs to the CC family of chemokines and is a ligand for CCR2. It is a well-known chemoattractant that has been found to be involved in the metastasis of various cancer types. It is expressed by endothelial cells, stromal cells, bone marrow osteoblast as well as by tumor cells [194,195]. Prostate cancer cells express higher amounts of CCL2, exhibiting both autocrine and paracrine functions [196]. Izhak et al. [197] revealed in their study that it is the predominantly expressed chemokine within primary tumors of prostate cancer patients and its production ultimately triggers a collapse of immunological tolerance to CCL2.

Tumor associated bone lesions are predominantly osteoblastic in nature, although osteolytic bone resorption may be present. In prostate cancer, CCL2 has been implicated as one of the main cytokines involved in tumor cell re-establishment in the bone marrow. The osteoclastogenesis potential of CCL2 is shown in a study wherein the levels of CCL2 expression in osteoblasts and endothelial cells were enhanced upon PC3 or VCaP cells inoculation into mice, with CCL2 promoting bone metastasis while also indirectly enhancing angiogenesis [133]. An in-vivo metastasis study in which CCL2 was overexpressed in PC3-luc cells demonstrated how CCL2 affects prostate cancer bone metastasis. PC3-luc tumors exhibiting elevated CCL2 expression manifested increased cancer growth and greater number of functional osteoclasts [134]. In another study to determine the role of CCL2/CCR2 axis in growth of prostate cancer in the bone, knocking down CCR2 abrogated tumor invasiveness [130]. Furthermore, in-vitro osteoclast formation and in vivo tumor volume were diminished upon transfecting prostate cancer cells with shRNA against CCL2 [130]. Loberg et al. [131] revealed the high secretion of CCL2 by human bone marrow endothelial (HBME) cells and their ability to chemoattract as well as induce metastasis of prostate cancer cells, via an AKT-dependent signaling mechanism, into the bone microenvironment. Again, the development of prostate cancer cells was found to be promoted through PTHrP-induced CCL2 production by osteoblasts and HBME cells [198]. The inhibition of CCL2 activity with neutralizing antibodies in an in-vivo model of prostate cancer metastasis decreased overall tumor burden [132].

In examining the role of CCL2 in modulating cell adhesion molecules, vis-à-vis their migratory potential, Lin et al. [135] showed that treatment of prostate cancer cells with CCL2 induced expression of αvβ3 integrin and inhibition of the CCL2-CCR2 signaling pathway decreased migration.

### 4.4. CXCL12/SDF-1α

CXCL12, also known as stromal-derived factor-1 (SDF-1α), is a member of the CXC family of chemokines that binds to CXCR4 and CXCR7 [199]. Expression of CXCL12 and CXCR4 are increased in prostate cancer, with high CXCR4 expression being an indicator for bone metastasis [109,200,201]. It is also secreted by stromal and endothelial cells. Taichman et al. [108] revealed that prostate cancer cell lines with metastatic origin from the bone tested positive for CXCR4 expression. Although the complete CXCL12/CXCR4-mediated molecular mechanisms through which prostate cancer cells re-establish themselves to the bone are still subject to further investigations, several probable mechanisms have, however, been proposed. Indeed, CXCL12 plays important roles in prostate tumor cell homing, re-establishment, and proliferation in metastatic sites through their modulatory effects on tumor adhesiveness and migration [202].

In a study to assess the role of the CXCL12/CXCR4 axis in prostate cancer migration and tumor invasiveness, it was reported that CXCL12 activation of prostate cancer cell lines, PC3 and LNCaP, increased their migratory potential via the upregulated expression of several metalloproteinases (MMPs) [203]. Similar findings were reported by Chinni et al. [204] who described enhanced migration and MMP9 secretion following exogenous CXCL12 stimulation of prostate cancer cells from bone tissue-derived conditioned media. Pharmacological blockage of the PI3K and MAP kinase pathways diminished this effect [204]. Immunohistochemical analysis of 148 prostatectomy patients revealed a correlation between CXCL12, VEGF, and MMP9 expression patterns and the appearance of lymph node metastatic carcinoma [205]. The study, therefore, concluded that CXCL12 expression level served as a predictor of prognosis for patients undergoing radical prostatectomy [205]. Another interesting study evaluating the regulatory role of CXCR4 in a mouse model of metastasis revealed decreased bone metastasis and VEGF and MMP9 expression, following knockdown of CXCR4 in PC3 cells [141].

The high levels of CXCL12 expressed in the bone microenvironment are indicative of its high affinity to home disseminating metastatic cell [139]. In fact, CXCL12 has been implicated in enhancement of prostate cancer cell metastasis to the bone. Stimulation by CXCL12 was found to promote prostate tumor migration across monolayers of bone marrow endothelial cells, increase invasion through basement membranes, as well as adhesiveness towards osteosarcomas [108]. The specific blockade of the CXCL12/CXCR4 axis in prostate cancer cells using hTERT promoter induced knockdown of CXCR4 decreased bone metastasis [201]. In another instance, a metastasis study in nude mice revealed a correlation between CXCL12 expression level and the organs of appearance of metastatic lesions [140]. Elevated CXCL12 as well as increased tumor metastasis were detected in skeletal tissues, whereas subsequent treatment with CXCR4 antibody decreased tumor spread [140].

Indeed, the CXCL12/CXCR4 axis has been shown to promote metastasis via modulation of cell adhesion molecule expression and integrin adhesiveness. In a recently reported study, CXCL12 stimulation increased the adhesiveness of the bone-derived metastatic cell lines (PC3 and C4-2B) to HBME cells via enhanced expression and activation of αvβ3 integrin receptors [142]. Despite the low levels of endogenous CXCR4 expression in LNCaP and DU-145 cells, it was found that CXCL12 facilitated increased adhesion of these prostate cancer cells to monolayers of endothelial cells and immobilized matrix through enhanced expression of α5 and β3 integrins [143]. Similar studies have also reported how upregulated α2β1 expression promotes prostate cancer bone metastasis [144,145].

The CXCL12/CXCR4 axis is also involved in enhancing angiogenesis in the tumor environment, and therefore, metastasis [136]. Prostate cancer cell lines overexpressing CXCR4 exhibited increased angiogenesis, characterized by enhanced microvascular density and functionality, as well as elevated metastasis to distant organs in a NOD/SCID mice xenograft model [137]. Inhibiting this effect by usage of a neutralizing antibody against CXCR4 diminished tumor size and intratumor blood vessel formation [137]. Similarly, blocking CXCR4 actions with an antagonist (CTCE-9908) in PC3-Bcl-2 cells in a xenograft model exhibited a decreased tumor size, which was associated with suppressed VEGF expression, angiogenesis, and lymphangiogenesis in the tumor microenvironment [138]. Apart from these, the enhanced activation of the CXCL12/CXCR4 axis in metastatic sites instituted an angiogenic switch and promoted prostate tumor metastasis that is mediated by a decreased expression of the glycolytic phosphoglycerate kinase 1 (PGK1) enzyme [206].

Furthermore, the role of CXCL12 in mediating stemness and neuroendocrine phenotypes to promote metastasis has recently been reported. Indeed, the overexpression of CXCL12γ promoted tumor metastasis and resistance to chemotherapy by inducing the transformation of human prostate cancer cells to a more cancer stem cell and neuroendocrine phenotype [207]. Blocking the CXCL12/CXCR4 axis via usage of a CXCR4 receptor antagonist (AMD3100) or antibody in prostate cancer cells, however, decreased tumor size and the population of progenitor cells [208].

Apart from all these, the involvement of the CXCL12/CXCR7 axis in metastasis of prostate cancer has also been documented in literature. Increased levels of expression of the atypical CXCR7 receptor is observed as prostate tumor progresses and becomes more aggressive, and higher levels of expression are also found in prostate cancer cell lines when compared to normal cells [209,210,211]. In evaluating the role of the CXCL12/CXCR7 axis in prostate cancer metastasis, Wang et al. [209], for example, described how overexpression of CXCR7 in human prostate cancer cell lines enhanced in vivo tumor growth and promoted metastasis via increased release of angiogenic cytokines, such as CXCL8 and VEGF. In another instance, migration and invasion was shown to be decreased following suppression of the CXCL12/CXCR7 axis in cell lines derived from an obesity-driven mouse model of Hi-Myc prostate cancer [212]. Indeed, knocking down CXCR7 in enzalutamide-resistant prostate cancer cells resulted in diminished invasiveness and tumor growth [211]. A similar phenomena was described by Luo et al. [213] wherein co-treatment of enzalutamide and a CXCR7 inhibitor significantly decreased migration, VEGF secretion, and tumor growth in castration-resistant C4-2B and VCaP cells.

### 4.5. RANKL

Receptor activator of NF-κB ligand (RANKL) is a member of the TNF family of cytokines. It has been extensively implicated for its role in remodeling of the bone microenvironment, with the RANKL/RANK/OPG axis actively involved in osteoclastogenesis and bone resorption within the skeletal system [149,152,214,215]. Interaction of RANKL with RANK initiates intracellular recruitment of TNF receptor-associated factors (TRAFs) as well as other adaptor proteins and ultimately leads to the activation of the MAPK, PI3K, and NFκB pathways [216]. RANKL exists either as a membrane-bound or soluble protein and is produced by bone marrow stromal, osteoblast, as well as T cells [217]. RANK on the other hand is expressed by diverse cells including tumor cells, immune cells, and osteoclast [149,214]. Penno et al. [218] reported surface membrane expression of RANKL in a number of prostate cancer cell lines, including PC3, LNCaP, DU-145, and whose expression was increased following their co-culture with human osteoblast-like cells (hoB).

RANKL is known to be involved in metastasis of various forms of cancer, including prostate cancer, to the bone. The suggestion of a correlation existing between the RANKL/RANK/OPG axis and metastatic prostate carcinoma was reported by Chen et al. [149], who described high expression of RANKL and its receptor (RANK) in metastatic cancer, with attendant higher prevalence of these proteins in bone metastasis as compared to lymph node. Christoph et al. [150] corroborated this finding using tissues obtained from radical prostatectomy patient and showed higher gene transcription of RANKL and RANK in those with bone metastasis. PC3 and DU-145 prostate cancer cell lines also express functionally active RANK receptor that induced phosphorylation of ERK1/2 and p38 upon agonist stimulation [118]. In addition, RANK-mediated activation of IκB kinase α (IKKα) inhibits maspin, a tumor suppressor, to promote prostate tumorigenesis, and the loss of function mutation of the IKKα gene in a TRAMP mouse model suppressed distant organ metastasis [151].

In-vitro activation of the RANKL/RANK pathway promoted increased metastatic potential and MMP-1 expression of the prostate cancer PC3 cell line, with an interesting decreased presence of osteoclastogenesis and osteolytic lesions following MMP-1 knockdown in a mouse model of metastasis [153]. Furthermore, Morrissey et al. [152] defined how the host-derived, and not tumor cell-derived, RANKL cytokine facilitates prostate tumor establishment and osteolysis within the bone by treating tumor-bearing animals with a human neutralizing antibody against tumor-secreted RANKL. A similar study in SCID mice, in which intratibially injected PC3 cells were used, demonstrated how the presence of malignancy enhanced levels of RANKL expression. Treatment of animals with a RANKL antagonist subsequently diminished tumor formation and bone lesion [148]. Other studies have also provided similar conclusions. For example, co-treatment of RANKL inhibitor osteoprotegerin (OPG) and docetaxel was found to significantly decrease tumor burden and osteolytic lesions in a murine model of prostate cancer bone metastasis [219], whereas sole treatment with OPG was reported to diminish the proportion of RANKL-positive osteoblasts and bone metastasis following castration of mice [220]. It may, therefore, be inferred that RANKL produced within the host metastatic sites are sufficient to initiate osteogenic changes and promote metastasis of tumor cells.

RANKL has also been shown to be involved in the reprogramming of tumor cells and EMT. In evaluating the involvement of RANKL in EMT, Odero-Marah et al. [146] identified a functionally active RANKL protein that was upregulated in the highly tumorigenic ARCaP cell line and which exhibited greater mesenchyme phenotype, osteoclastogenesis, and bone spread, when compared to normal ARCaP cells. In a different study, the stimulation of the RANKL/RANK or c-Met pathway was found to promote activation of transcription factors related to stem cell-like properties, neuroendocrine differentiation, osteomimicry, and EMT in prostate cancer cells [147]. Apart from this, it was also revealed in the same study that metastatic RANKL-expressing LNCaP cells had the ability to reprogram and transform naïve LNCaP cells to elicit a metastatic phenotype, when co-injected in a metastatic mouse model system [147].

### 4.6. CXCL8/IL-8

CXCL8 is an ELR-positive pro-inflammatory protein that belongs to the CXC family of chemokines and binds to two homologous GPCRs known as CXCR1 and CXCR2 [221]. Elevated CXCL8 expression is observed in prostate cancer tissues compared with paired normal controls, as well as in prostate cancer cell lines, and its activation enhances their migratory and invasive potential [222]. Lehrer et al. [223] revealed significantly increased serum CXCL8 production in prostate cancer patients with bone metastasis. Increased CXCL8 expression, with attendant MMP9 expression was observed in the more metastatic PC3 and DU-145 cells relative to the less metastatic LNCaP cell line [88]. Similarly, Murphy et al. [224] reported the correlation of CXCL8, CXCR1, and CXCR2 expression in prostate cancer with advancing disease stage and its ability in promotion angiogenesis.

CXCL8 effects on prostate cancer metastasis are mediated mainly via its proangiogenic ability within tumors as well as its influence on EMT and these have been documented by various studies. For example, CXCL8 expression was previously shown in an in vivo study to correlate with increased angiogenesis, tumor development, and metastasis in human prostate cancer cells [155]. There appears to be a positive correlation between transcriptional expression of angiogenic factors (including CXCL8) and metastatic prostate cancer [88]. Inoue et al. [156] described how CXCL8 overexpression in human PC3 cells in an orthotopic nude mouse model enhanced tumor growth, angiogenesis, and metastasis via upregulated MMP9 expression and collagenase activity. Tumors from CXCL8 overexpressing LNCaP cells exhibited increased tumor size, vasculature, and microvessel formation when compared to control cells, with CXCL8 overexpressing LNCaP cells also exhibiting enhanced invasiveness and MMP9 expression [225]. Indeed, CXCL8 activation is capable of transactivating the VEGFR2 receptor to induce endothelial permeability and thereby promote angiogenesis [157].

The CXCL8 signaling pathway has similarly been implicated in AR expression and regulation. In one instance, increased CXCL8 expression has been linked with markedly diminished AR levels and the generation of a more aggressive disease in both primary as well as metastatic prostate cancer [226]. In prostate cancer cell lines, distant metastases and PDX lines, detectable levels of CXCL8 have been observed [226]. Apart from these, recent studies have been focused on evaluation of the role of the CXCL8/CXCR2 axis in NE phenotypes and cells vis-a-vis metastasis. This is because neuroendocrine cells have found new relevance in development of metastatic and drug-resistant prostate cancer. In small cell prostate cancer, for instance, NE cells are highly metastatic and resistant to treatment [227]. In a recent study, Li et al. [154] reported how CXCR2 expression is associated with prostate cancer progression and tumor grade and described how its blockade may serve as a viable approach to overcome the challenges of treating advanced therapy-resistant and metastatic prostate cancers. The study further revealed NE cells as being positive for CXCR2 and CXCL8 expression and alluded to their involvement in EM remodeling, angiogenesis, and invasion. NE phenotype causes cellular switch to a form that exhibits high enrichment for gene sets of EMT, tumorigenesis, angiogenesis, and stem cell markers [154].

CXCL8 can also induce osteoclastogenesis and bone resorption. Lu et al. [228] revealed how human bone marrow mononuclear cells (HBMC) were differentiated to osteoclast-like cells following stimulation by CXCL8, obtained from PC3-conditioned medium. IL8 stimulation in the absence of RANKL also induced dental slices bone resorption [228].

### 4.7. CX3CL1

Endothelial cells and osteoblasts are known to express CX3CL1 (fractalkine) as a transmembrane protein. Therefore, cells that express its receptor, CX3CR1, are able to adhere to endothelial cells and extravasate to metastatic sites. CX3CL1 has been reported to promote metastasis of different tumor types [229,230,231], and CX3CR1 has been found to be overexpressed in prostate cancer tissues with spinal metastasis [232]. The actions of the CX3CL1/CX3CR1 axis in prostate tumor metastasis are mediated via induction of EMT and promotion of cell migration.

Human prostate tumors express CX3CR1, which facilitates their adhesion to bone marrow CX3CL1-expressing endothelial cells as well as osteoblasts, and triggers PI3K/AKT pathway activation [233]. Furthermore, Jamieson et al. [234] reported increased levels of CX3CR1 expression in malignant prostate tissues and the presence of a soluble form of fractalkine in bone marrow supernatants. This soluble form can be cleaved off from bone cell membranes, and not bone marrow endothelial cells, in an androgen-dependent manner [234]. The ability of CX3CL1 to induce enhanced invasiveness and EMT was recently reported. Tang et al. [76] in their study described how CX3CL1 induced EMT and promoted tumor cell migration and invasion in the PC3 and DU-145 prostate cancer cell lines.

### 4.8. VEGF

Numerous studies have evaluated the involvement of VEGF in the various transitional stages of prostate cancer spread to end-organs, chiefly the bone. VEGF expression is raised in prostate cancer cells, relative to benign prostatic hyperplasia (BPH) and normal tissues [235]. Similar observation was reported in a retrospective study conducted by Green et al. [236], in which elevated VEGF levels was found to be correlated with disease prognosis. Prostate tumor cells express VEGF and its receptors (VEGFRs), and the increased migration of metastatic cells was reported to be regulated by the VEGF/VEGFR-2 axis [237]. Other studies have equally shown how plasma serum levels of VEGF are increased in prostate cancer [238], with metastatic patients exhibiting higher production levels [239].

VEGF typically exerts its tumor microvasculature remodeling action by stimulating the growth and proliferation of endothelial cells as well as altering the permeability of blood vessels. The consequence of these is increased tumor cell invasiveness and enhanced potential to metastasize. Indeed, the neovascularization and tumor proliferation observed in prostate cancer is induced via the activation of the VEGF/VEGFR axis, and this is facilitated either through autocrine or paracrine mechanisms [240]. For example, in a recent study by Montecinos et al. [158], a surge in AR-induced VEGF expression was shown to precede increased angiogenesis of human vasculature in a nude mice xenograft of implanted human prostate tissues. Using orthotopic and metastatic prostate cancer mouse models, inhibition of the VEGF/VEGFR axis by an antibody (DC101) against the VEGF receptor, flk-1, reduced tumor-induced angiogenesis and suppressed metastasis, and this was associated with diminished MMP9 production by endothelial cells [159]. Furthermore, VEGF pathway activation was found to enhance migration of the metastatic LNCaP-C4-2 cell line to stimulation by fibronectin and bone sialoprotein [237]. Treatment with the VEGF inhibitor, Bevacizumab, suppressed metastatic C4-2B prostate cancer cell angiogenesis and invasion in an in-vitro experiment [241].

VEGF has also been shown to induce osteoblastic activity in bone tissues [160,161]. Within bone microenvironment, the VEGF/VEGFR axis is involved in creation of the premetastatic niche, remodeling of the bone, and its recognition by the metastatic tumor cells [87,107]. In a metastatic mice model, treatment of animal with a VEGFR inhibitor (PTK787) following an intratibial injection of C4-2B cells was found to result in diminished tumor burden and bone mineral density as well as altered bone lesions when compared to control mice [161]. Indeed Dai et al. [160] described how osteoblastogenesis, induced by bone morphogenetic protein (BMP), is mediated through the VEGF/VEGFR axis to promote prostate cancer metastasis. Equally important is how VEGF stimulation modulates the expression of cell adhesion molecules as tumor cells transit into metastatic sites. As described by De et al. [162], the VEGF/VEGFR axis via an autocrine loop activates αvβ3 and αvβ5 integrins on prostate cancer cells, which allows the tumor cells to migrate into bone tissues in a SPARC animal model. The resultant effect of this is further upregulation of VEGF expression within the bone nexus and vascular angiogenesis to promote tumor metastasis [162]. This study also reported the co-localization of VEGFR-2 with activated integrins in metastatic prostate tumor tissues [162]. VEGF may also exert suppressive actions on the immune cells [242].

### 4.9. IL-1

IL-1 is a cytokine involved in immunity and inflammation. IL-1β is upregulated in prostate cancer, and its expression correlates with Gleason scores ≥ 7 [243]. Using a mouse model, Liu et al. [243] showed the ability of nonmetastatic cancer cells to grow in bone lesions when IL-1β is overexpressed, while its knockdown in highly metastatic cells resulted in diminished growth within the bone. In fact, IL-1β was described as promoting a neuroendocrine phenotype, as its expression inversely correlates with AR levels [243,244]. This was further confirmed by Thomas-Jardin et al. [245], who reported IL-1 reprogramming of AR-positive prostate cancer cells to exhibit AR-negative phenotype through its suppression of AR mediated genes. IL-1 has also been implicated in bone osteoclastogenesis promotion. Using a SCID mice metastasis model of prostate cancer, IL-1 was revealed as one of the cytokines associated with osteoclastogenesis and with attendant metastasis promotion ability [163].

### 4.10. CXCL1

CXCL1 is upregulated in prostate cancer [246]. It promotes prostate cell EMT, migration, and invasion via AKT/NFκB axis [164]. Lu et al. [165] recently reported how CXCL1 triggers EMT, migration, and prostate tumor progression via the CXCL1-LCN2 paracrine axis. The role of CXCL1 in osteoclast development has also been identified. In metastatic prostate cancer, the maturation of osteoclast is quickened following CXCL1 stimulation, and this action has been shown to be blocked upon treatment with neutralizing antibodies against CXCL1 [247].

### 4.11. IL-7

The level of IL-7 expression has been suggested as impacting the rate of survival of patients with prostate cancer, and upregulated expression of both IL-7 and its receptor, IL-7R, have been detected in prostate tumor cells [248,249]. In fact, increased serum levels of IL-7 are observed during the early stage of prostate tumorigenesis, compared to BPH [250]. Inhibition of the IL-7/IL-7R axis decreases prostate cancer cell invasion, migration, as well as MMP3 and MMP7 expression via suppressive effects on both the AKT and NFκB pathways [249]. Seol et al. [77] reported how overexpressing IL-7Rα in PC3 cells, via usage of a lentiviral delivery system, enhanced prostate cancer metastasis to the bone in a murine model of metastasis. Similarly, the activation of the IL-7/IL-7R axis was found to promote tumor cell mesenchyma switch, migration, and invasion in-vitro, thereby showing that IL-7 stimulation can promote EMT and metastasis [77].

### 4.12. CXCL16

CXCL16 and its receptor, CXCR6, have been found to possess functional roles in various stages of prostate cancer metastasis including EMT, invasion, and tumor cell homing to secondary sites. Multiple studies have reported increased expression of both CXCL16 and CXCR6 in advanced stages of prostate cancer as well as in metastatic tissues, with expression pattern correlating with Gleason score of patients [251,252,253,254]. Interestingly, patients presenting with significantly enhanced expression of these proteins exhibit poor disease prognosis [255]. As alluded in a recent study, activation of the CXCL16/CXCR6 pathway promoted increased migration and invasion of prostate cancer cells via its ability to induce cytoskeletal protein reorganization through enhanced Ezrin phosphorylation and clustering of αvβ3 integrin structures [168]. Noteworthy is that stimulation of the CXCR6 pathway enhanced prostate tumor cell adhesion to endothelial cells and increased MMP expression [168]. This, therefore, portrays the ability of CXCL16 to promote extravasation of migrating tumor cells. Hu et al. [167] further suggested the modulatory role of the CXCL16/CXCR6 axis in metastasis of prostate cancer to skeletal tissues, as is typically observed with CXCL12, and showed how CXCL16 secreted by bone cells can attract prostate tumors into bone structures. Their study also revealed increased invasion and MMP upregulation, following activation of CXCR6 [167]. Furthermore, the involvement of cancer-associated fibroblasts (CAFs) in tumorigenesis cannot be understated. It is now known that CXCL16 secreted by prostate tumor cells are capable of recruiting mesenchymal stem cells to TME and promoting their transition to become CAFs [166]. The resultant effect of this action is the consequential release of CXCL12 by the CAFs to facilitate metastasis via induction of EMT in the prostate cancer cells [166].

## 5. Conclusions

Metastatic prostate cancer remains a major healthcare problem and represents the main disease associated cause of death in prostate cancer patients. The bone constitutes the primary site of metastasis; even with the ability of prostate tumors to metastasize to the lymph nodes, lungs, brain, and liver tissue [15,16,17,18]. Although the development of this end-stage of prostate cancer disease involves a convoluted interplay and cross talk between various cells (tumor cells, stromal cells, immune cells, adipocytes, and endothelial cells) and secreted factors (cytokines, chemokines, and growth factors), the modulatory roles of cytokines and chemokines remains highly important in the sequence of events that drive metastasis. In prostate cancer metastasis, it is interesting to note the associated involvement of numerous cytokines and chemokines in the process of ECM remodeling, EMT, angiogenesis, intravasation, premetastatic niche creation, extravasation, establishment, and development of escaped tumor cells as well as remodeling of the metastatic TME. More important is the fact that the advancement of prostate cancer disease and development of metastasis has also been associated with upregulated levels of expression of various cytokines and their receptors, as well as dysregulation of their signaling axis.

During the early phase of metastasis, cytokines such as TGFβ, IL-6, CXCL8, IL-7, CXCL16, and CX3CL1 induce EMT in prostate cancer cells and transforms them to exhibit higher migratory and invasive potentials [76,77,80,81,122]. This is achieved by signal-mediated rearrangement of actin cytoskeleton that promotes migratory protrusion formation in tumor cells and upregulated transcription of genes related to mesenchymal and stemness phenotypes. Moreover, CXCL12, CXCL8, or RANKL released into TME have been found capable of upregulating MMP production and breaking down ECM to induce increased tumor cell invasiveness [153,156,203,204]. Furthermore, metastasis requires the occurrence of the angiogenic switch, wherein vascularization and endothelial proliferation is increased within the tumor. Proangiogenic cytokines such as VEGF, CXCL8, IL-6, TGFβ, and CXCL12 drive this process, although the VEGF/VEGFR axis is the main culprit involved in promotion of angiogenesis [83,85,89]. Increased blood innervation and oxygenation of the TME consequently allows for increased escape of tumor cells into the circulation and transportation to distal organs. This enhanced angiogenesis is also needed for establishment of metastatic cells to secondary sites. Other than these, CCL2 and CXCL12 also play modulatory roles in promoting the expression of adhesion molecules, such as integrins, during metastasis and with a concomitant effect of enhancing arrest of CTCs to endothelial cells before homing. Finally, the involvement of cytokines such as CXCL12, CCL2, RANKL, IL-6, VEGF, and TGFβ in formation of the premetastatic niche, endothelial arrest of CTCs, extravasation, remodeling of the new TME, and establishment of viable macrometastases have been extensively studied [102,103]. For instance, in skeletal tissues, the increased production of CXCL12 during the metastatic process facilitate a concentration gradient-dependent migration of CTCs into the bone microenvironment. Furthermore, other cytokines such as CCL2, RANKL, IL-6, CXCL8, and IL-1 are known to drive osteoclastogenesis and bone resorption: key events observed following prostate tumor metastasis to the bone [113,114,115,133,219].

In summary, the consequences of prostate cancer metastasis remain grave and the process of its development is influenced by a multitude of factors. The presented knowledge in this review, therefore, provides a wealth of understanding about how cytokines and chemokines help drive prostate cancer metastasis. The various cytokine-receptor axes are not only involved in maintenance of normal homeostasis within the human body but their aberrant expression and signaling transduction also, in fact, do constitute key elements driving metastasis of various tumor types, prostate cancer inclusive.

## Figures and Tables

**Table 1:** Cytokines and chemokines involvement in different stages of the metastatic process of prostate cancer.

Cytokine	Receptor	Effects during Prostate Cancer Metastasis	References
TGFβ	TGFβR	EMT	[79,80,120,121,122]
		Angiogenesis	[95,96]
		Homing and establishment of metastasis	[123,124]
IL-6	IL-6R	EMT	[81,125]
		Angiogenesis	[85]
		Homing and establishment of metastasis	[126,127]
		Remodeling of metastatic site	[128,129]
CCL2	CCR2	Homing and establishment of metastasis	[130,131,132]
		Remodeling of metastatic site	[133,134]
		Regulation of Integrin expression	[135]
CXCL12	CXCR4	Angiogenesis	[136,137,138]
	CXCR7	Homing and establishment of metastasis	[108,109,110,139,140,141]
		Regulation of Integrin expression	[142,143,144,145]
RANKL	RANK	EMT	[146,147]
		Homing and establishment of metastasis	[148,149,150,151,152]
		Remodeling of metastatic site	[119,153]
CXCL8	CXCR1	EMT	[154]
	CXCR2	Angiogenesis	[155,156,157]
CX3CL1	CX3CR1	EMT	[76]
VEGF	VEGFR	Angiogenesis	[91,92,93,94,158,159]
		Homing and establishment of metastasis	[160,161]
		Regulation of integrin expression	[162]
IL-1	IL-1R	Promotes invasion and metastasis	[163]
CXCL1	CXCR1	EMT	[164,165]
	CXCR2		
IL-7	IL-7R	EMT	[77]
CXCL16	CXCR6	EMT	[166]
		Promotes invasion and metastasis	[167,168]

## Abbreviations

ADT	Androgen deprivation therapy
AR	Androgen receptor
BPH	Benign prostatic hyperplasia
CRPC	Castration-resistant prostate cancer
CTCs	Circulating tumor cells
ECM	Extracellular matrix
EMT	Epithelial-mesenchymal-transition
GPCRs	G-protein coupled receptors
MET	Mesenchymal-epithelial-transition
MMP	Metalloproteinases
NE	Neuroendocrine
OPG	Osteoprotegerin
PINs	Prostatic intraepithelial neoplasia
RANKL	Receptor activator of nuclear factor kappa B ligand
TGFβ	Transforming growth factor beta
TNF	Tumor necrosis factor
VEGF	Vascular endothelial growth factor

## References

[1] Shen M.M., Abate-Shen C. (2010). Molecular genetics of prostate cancer: New prospects for old challenges. Genes Dev..

[2] Feldman B.J., Feldman D. (2001). The development of androgen-independent prostate cancer. Nat. Rev. Cancer.

[3] Litwin M.S., Tan H.J. (2017). The Diagnosis and Treatment of Prostate Cancer: A Review. JAMA.

[4] Karantanos T., Corn P.G., Thompson T.C. (2013). Prostate cancer progression after androgen deprivation therapy: Mechanisms of castrate resistance and novel therapeutic approaches. Oncogene.

[5] Wang Q., Li W., Zhang Y., Yuan X., Xu K., Yu J., Chen Z., Beroukhim R., Wang H., Lupien M. (2009). Androgen receptor regulates a distinct transcription program in androgen-independent prostate cancer. Cell.

[6] Wang G., Zhao D., Spring D.J., DePinho R.A. (2018). Genetics and biology of prostate cancer. Genes Dev..

[7] Sartor O., de Bono J.S. (2018). Metastatic Prostate Cancer. N. Engl. J. Med..

[8] Teo M.Y., Rathkopf D.E., Kantoff P. (2019). Treatment of Advanced Prostate Cancer. Annu. Rev. Med..

[9] Heinlein C., Chang C. (2004). Androgen receptor in prostate cancer. Endocr. Rev..

[10] Bluemn E.G., Coleman I.M., Lucas J.M., Coleman R.T., Hernandez-Lopez S., Tharakan R., Bianchi-Frias D., Dumpit R.F., Kaipainen A., Corella A.N. (2017). Androgen Receptor Pathway-Independent Prostate Cancer Is Sustained through FGF Signaling. Cancer Cell.

[11] Adekoya T., Smith N., Aladeniyi T., Blumer J., Chen X., Richardson R. (2019). Activator of G protein signaling 3 modulates prostate tumor development and progression. Carcinogenesis.

[12] Pienta K.J., Bradley D. (2006). Mechanisms underlying the development of androgen-independent prostate cancer. Clin. Cancer Res..

[13] Ghosh P., Malik S., Bedolla R., Wang Y., Mikhailova M., Prihoda T., Troyer D., Kreisberg J. (2005). Signal transduction pathways in androgen-dependent and -independent prostate cancer cell proliferation. Endocr. Relat. Cancer.

[14] Tangen C.M., Faulkner J.R., Crawford E.D., Thompson I.M., Hirano D., Eisenberger M., Hussain M. (2003). Ten-year survival in patients with metastatic prostate cancer. Clin. Prostate Cancer.

[15] Logothetis C.J., Lin S.H. (2005). Osteoblasts in prostate cancer metastasis to bone. Nat. Rev. Cancer.

[16] Jin J.K., Dayyani F., Gallick G.E. (2011). Steps in prostate cancer progression that lead to bone metastasis. Int. J. Cancer.

[17] Datta K., Muders M., Zhang H., Tindall D.J. (2010). Mechanism of lymph node metastasis in prostate cancer. Future Oncol..

[18] Vinjamoori A.H., Jagannathan J.P., Shinagare A.B., Taplin M.E., Oh W.K., Van den Abbeele A.D., Ramaiya N.H. (2012). Atypical metastases from prostate cancer: 10-year experience at a single institution. AJR Am. J. Roentgenol..

[19] Testa U., Castelli G., Pelosi E. (2019). Cellular and Molecular Mechanisms Underlying Prostate Cancer Development: Therapeutic Implications. Medicines.

[20] Taichman R.S., Loberg R.D., Mehra R., Pienta K.J. (2007). The evolving biology and treatment of prostate cancer. J. Clin. Investig..

[21] Berish R.B., Ali A.N., Telmer P.G., Ronald J.A., Leong H.S. (2018). Translational models of prostate cancer bone metastasis. Nat. Rev. Urol..

[22] Luzzi K.J., MacDonald I.C., Schmidt E.E., Kerkvliet N., Morris V.L., Chambers A.F., Groom A.C. (1998). Multistep nature of metastatic inefficiency: Dormancy of solitary cells after successful extravasation and limited survival of early micrometastases. Am. J. Pathol..

[23] Tisoncik J., Korth M., Simmons C., Farrar J., Martin T., Katze M. (2012). Into the Eye of the Cytokine Storm. Microbiol. Mol. Biol. Rev..

[24] Dranoff G. (2004). Cytokines in cancer pathogenesis and cancer therapy. Nat. Rev. Cancer.

[25] Kishimoto T., Taga T., Akira S. (1994). Cytokine signal transduction. Cell.

[26] Ozaki K., Leonard W.J. (2002). Cytokine and cytokine receptor pleiotropy and redundancy. J. Biol. Chem..

[27] Zhang J.M., An J. (2007). Cytokines, inflammation, and pain. Int. Anesthesiol. Clin..

[28] Lee S., Margolin K. (2011). Cytokines in cancer immunotherapy. Cancers.

[29] Altan-Bonnet G., Mukherjee R. (2019). Cytokine-mediated communication: A quantitative appraisal of immune complexity. Nat. Rev. Immunol..

[30] Borish L.C., Steinke J.W. (2003). 2. Cytokines and chemokines. J. Allergy Clin. Immunol..

[31] Leonard W.J., Lin J.X. (2000). Cytokine receptor signaling pathways. J. Allergy Clin. Immunol..

[32] Rawlings J.S., Rosler K.M., Harrison D.A. (2004). The JAK/STAT signaling pathway. J. Cell Sci..

[33] Platanias L.C. (2005). Mechanisms of type-I- and type-II-interferon-mediated signalling. Nat. Rev. Immunol..

[34] Watowich S.S., Wu H., Socolovsky M., Klingmuller U., Constantinescu S.N., Lodish H.F. (1996). Cytokine receptor signal transduction and the control of hematopoietic cell development. Annu. Rev. Cell Dev. Biol..

[35] Steelman L.S., Pohnert S.C., Shelton J.G., Franklin R.A., Bertrand F.E., McCubrey J.A. (2004). JAK/STAT, Raf/MEK/ERK, PI3K/Akt and BCR-ABL in cell cycle progression and leukemogenesis. Leukemia.

[36] Charo I.F., Ransohoff R.M. (2006). The many roles of chemokines and chemokine receptors in inflammation. N. Engl. J. Med..

[37] Griffith J.W., Sokol C.L., Luster A.D. (2014). Chemokines and chemokine receptors: Positioning cells for host defense and immunity. Annu. Rev. Immunol..

[38] Zlotnik A., Yoshie O. (2012). The chemokine superfamily revisited. Immunity.

[39] Salanga C.L., Handel T.M. (2011). Chemokine oligomerization and interactions with receptors and glycosaminoglycans: The role of structural dynamics in function. Exp. Cell Res..

[40] Nasser M.W., Raghuwanshi S.K., Grant D.J., Jala V.R., Rajarathnam K., Richardson R.M. (2009). Differential activation and regulation of CXCR1 and CXCR2 by CXCL8 monomer and dimer. J. Immunol..

[41] Fernandez E.J., Lolis E. (2002). Structure, function, and inhibition of chemokines. Annu. Rev. Pharmacol. Toxicol..

[42] Mellado M., Rodríguez-Frade J.M., Mañes S. (2001). Martínez-AC: Chemokine signaling and functional responses: The role of receptor dimerization and TK pathway activation. Annu. Rev. Immunol..

[43] Chow M.T., Luster A.D. (2014). Chemokines in cancer. Cancer Immunol. Res..

[44] Nagarsheth N., Wicha M.S., Zou W. (2017). Chemokines in the cancer microenvironment and their relevance in cancer immunotherapy. Nat. Rev. Immunol..

[45] Cabioglu N., Gong Y., Islam R., Broglio K.R., Sneige N., Sahin A., Gonzalez-Angulo A.M., Morandi P., Bucana C., Hortobagyi G.N. (2007). Expression of growth factor and chemokine receptors: New insights in the biology of inflammatory breast cancer. Ann. Oncol..

[46] Kim J., Takeuchi H., Lam S.T., Turner R.R., Wang H.J., Kuo C., Foshag L., Bilchik A.J., Hoon D.S. (2005). Chemokine receptor CXCR4 expression in colorectal cancer patients increases the risk for recurrence and for poor survival. J. Clin. Oncol..

[47] Culig Z. (2011). Cytokine disbalance in common human cancers. Biochim. Biophys. Acta.

[48] Balkwill F. (2004). Cancer and the chemokine network. Nat. Rev. Cancer.

[49] Chen Z., Malhotra P.S., Thomas G.R., Ondrey F.G., Duffey D.C., Smith C.W., Enamorado I., Yeh N.T., Kroog G.S., Rudy S. (1999). Expression of proinflammatory and proangiogenic cytokines in patients with head and neck cancer. Clin. Cancer Res..

[50] Vindrieux D., Escobar P., Lazennec G. (2009). Emerging roles of chemokines in prostate cancer. Endocr. Relat. Cancer.

[51] Akashi T., Koizumi K., Tsuneyama K., Saiki I., Takano Y., Fuse H. (2008). Chemokine receptor CXCR4 expression and prognosis in patients with metastatic prostate cancer. Cancer Sci..

[52] Brown J.M., Corey E., Lee Z.D., True L.D., Yun T.J., Tondravi M., Vessella R.L. (2001). Osteoprotegerin and rank ligand expression in prostate cancer. Urology.

[53] Jennbacken K., Vallbo C., Wang W., Damber J.E. (2005). Expression of vascular endothelial growth factor C (VEGF-C) and VEGF receptor-3 in human prostate cancer is associated with regional lymph node metastasis. Prostate.

[54] Seyfried T.N., Huysentruyt L.C. (2013). On the origin of cancer metastasis. Crit. Rev. Oncog..

[55] Guan X. (2015). Cancer metastases: Challenges and opportunities. Acta Pharm. Sin. B.

[56] Gupta G.P., Massagué J. (2006). Cancer metastasis: Building a framework. Cell.

[57] Semenas J., Allegrucci C., Boorjian S.A., Mongan N.P., Persson J.L. (2012). Overcoming drug resistance and treating advanced prostate cancer. Curr. Drug Targets.

[58] Khan M.I., Hamid A., Adhami V.M., Lall R.K., Mukhtar H. (2015). Role of epithelial mesenchymal transition in prostate tumorigenesis. Curr. Pharm. Des..

[59] Thiery J.P., Acloque H., Huang R.Y., Nieto M.A. (2009). Epithelial-mesenchymal transitions in development and disease. Cell.

[60] Sistigu A., Di Modugno F., Manic G., Nisticò P. (2017). Deciphering the loop of epithelial-mesenchymal transition, inflammatory cytokines and cancer immunoediting. Cytokine Growth Factor Rev..

[61] Fares J., Fares M.Y., Khachfe H.H., Salhab H.A., Fares Y. (2020). Molecular principles of metastasis: A hallmark of cancer revisited. Signal. Transduct. Target. Ther..

[62] Pastushenko I., Brisebarre A., Sifrim A., Fioramonti M., Revenco T., Boumahdi S., Van Keymeulen A., Brown D., Moers V., Lemaire S. (2018). Identification of the tumour transition states occurring during EMT. Nature.

[63] Jolly M.K., Tripathi S.C., Jia D., Mooney S.M., Celiktas M., Hanash S.M., Mani S.A., Pienta K.J., Ben-Jacob E., Levine H. (2016). Stability of the hybrid epithelial/mesenchymal phenotype. Oncotarget.

[64] Ye X., Weinberg R.A. (2015). Epithelial-Mesenchymal Plasticity: A Central Regulator of Cancer Progression. Trends Cell Biol..

[65] Mani S.A., Guo W., Liao M.J., Eaton E.N., Ayyanan A., Zhou A.Y., Brooks M., Reinhard F., Zhang C.C., Shipitsin M. (2008). The epithelial-mesenchymal transition generates cells with properties of stem cells. Cell.

[66] Armstrong A.J., Marengo M.S., Oltean S., Kemeny G., Bitting R.L., Turnbull J.D., Herold C.I., Marcom P.K., George D.J., Garcia-Blanco M.A. (2011). Circulating tumor cells from patients with advanced prostate and breast cancer display both epithelial and mesenchymal markers. Mol. Cancer Res..

[67] Figiel S., Vasseur C., Bruyere F., Rozet F., Maheo K. (2017). Fromont G: Clinical significance of epithelial-mesenchymal transition markers in prostate cancer. Hum. Pathol.

[68] Lang S.H., Hyde C., Reid I.N., Hitchcock I.S., Hart C.A., Bryden A.A., Villette J.M., Stower M.J., Maitland N.J. (2002). Enhanced expression of vimentin in motile prostate cell lines and in poorly differentiated and metastatic prostate carcinoma. Prostate.

[69] Umbas R., Schalken J.A., Aalders T.W., Carter B.S., Karthaus H.F., Schaafsma H.E., Debruyne F.M., Isaacs W.B. (1992). Expression of the cellular adhesion molecule E-cadherin is reduced or absent in high-grade prostate cancer. Cancer Res..

[70] Sethi S., Macoska J., Chen W., Sarkar F.H. (2010). Molecular signature of epithelial-mesenchymal transition (EMT) in human prostate cancer bone metastasis. Am. J. Transl. Res..

[71] Stylianou N., Lehman M.L., Wang C., Fard A.T., Rockstroh A., Fazli L., Jovanovic L., Ward M., Sadowski M.C., Kashyap A.S. (2019). A molecular portrait of epithelial-mesenchymal plasticity in prostate cancer associated with clinical outcome. Oncogene.

[72] Tanaka H., Kono E., Tran C.P., Miyazaki H., Yamashiro J., Shimomura T., Fazli L., Wada R., Huang J., Vessella R.L. (2010). Monoclonal antibody targeting of N-cadherin inhibits prostate cancer growth, metastasis and castration resistance. Nat. Med..

[73] Nauseef J.T., Henry M.D. (2011). Epithelial-to-mesenchymal transition in prostate cancer: Paradigm or puzzle?. Nat. Rev. Urol..

[74] David J.M., Dominguez C., Hamilton D.H., Palena C. (2016). The IL-8/IL-8R Axis: A Double Agent in Tumor Immune Resistance. Vaccines.

[75] Wang H., Fang R., Wang X.F., Zhang F., Chen D.Y., Zhou B., Wang H.S., Cai S.H., Du J. (2013). Stabilization of Snail through AKT/GSK-3β signaling pathway is required for TNF-α-induced epithelial-mesenchymal transition in prostate cancer PC3 cells. Eur. J. Pharmacol..

[76] Tang J., Xiao L., Cui R., Li D., Zheng X., Zhu L., Sun H., Pan Y., Du Y., Yu X. (2016). CX3CL1 increases invasiveness and metastasis by promoting epithelial-to-mesenchymal transition through the TACE/TGF-α/EGFR pathway in hypoxic androgen-independent prostate cancer cells. Oncol. Rep..

[77] Seol M.A., Kim J.H., Oh K., Kim G., Seo M.W., Shin Y.K., Sim J.H., Shin H.M., Seo B.Y., Lee D.S. (2019). Interleukin-7 Contributes to the Invasiveness of Prostate Cancer Cells by Promoting Epithelial-Mesenchymal Transition. Sci. Rep..

[78] Zhau H.E., Odero-Marah V., Lue H.W., Nomura T., Wang R., Chu G., Liu Z.R., Zhou B.P., Huang W.C., Chung L.W. (2008). Epithelial to mesenchymal transition (EMT) in human prostate cancer: Lessons learned from ARCaP model. Clin. Exp. Metastasis.

[79] Chen X.H., Liu Z.C., Zhang G., Wei W., Wang X.X., Wang H., Ke H.P., Zhang F., Wang H.S., Cai S.H. (2015). TGF-β and EGF induced HLA-I downregulation is associated with epithelial-mesenchymal transition (EMT) through upregulation of snail in prostate cancer cells. Mol. Immunol..

[80] Sun Y., Schaar A., Sukumaran P., Dhasarathy A., Singh B.B. (2018). TGFβ-induced epithelial-to-mesenchymal transition in prostate cancer cells is mediated via TRPM7 expression. Mol. Carcinog.

[81] Giannoni E., Bianchini F., Masieri L., Serni S., Torre E., Calorini L., Chiarugi P. (2010). Reciprocal activation of prostate cancer cells and cancer-associated fibroblasts stimulates epithelial-mesenchymal transition and cancer stemness. Cancer Res..

[82] Stifter S., Dorđević G. (2014). Prostate cancer and new insights in angiogenesis. Front. Oncol..

[83] Yang K.Q., Liu Y., Huang Q.H., Mo N., Zhang Q.Y., Meng Q.G., Cheng J.W. (2017). Bone marrow-derived mesenchymal stem cells induced by inflammatory cytokines produce angiogenetic factors and promote prostate cancer growth. BMC Cancer.

[84] Rajabi M., Mousa S.A. (2017). The Role of Angiogenesis in Cancer Treatment. Biomedicines.

[85] Nicholson B., Theodorescu D. (2004). Angiogenesis and prostate cancer tumor growth. J. Cell Biochem..

[86] Russo G., Mischi M., Scheepens W., De la Rosette J.J., Wijkstra H. (2012). Angiogenesis in prostate cancer: Onset, progression and imaging. BJU Int..

[87] Roberts E., Cossigny D.A., Quan G.M. (2013). The role of vascular endothelial growth factor in metastatic prostate cancer to the skeleton. Prostate Cancer.

[88] Aalinkeel R., Nair M.P., Sufrin G., Mahajan S.D., Chadha K.C., Chawda R.P., Schwartz S.A. (2004). Gene expression of angiogenic factors correlates with metastatic potential of prostate cancer cells. Cancer Res..

[89] Hrouda D., Nicol D.L., Gardiner R.A. (2003). The role of angiogenesis in prostate development and the pathogenesis of prostate cancer. Urol. Res..

[90] Dall’Era M.A., Shih S.J., Yang J., Benik S., Gandour-Edwards R., Evans C.P. (2001). Differential expression of angiogenic cytokines by cell lines and primary cultures of human prostate cancer. Prostate Cancer Prostatic Dis..

[91] Melnyk O., Zimmerman M., Kim K.J., Shuman M. (1999). Neutralizing anti-vascular endothelial growth factor antibody inhibits further growth of established prostate cancer and metastases in a pre-clinical model. J. Urol..

[92] Borgström P., Bourdon M.A., Hillan K.J., Sriramarao P., Ferrara N. (1998). Neutralizing anti-vascular endothelial growth factor antibody completely inhibits angiogenesis and growth of human prostate carcinoma micro tumors in vivo. Prostate.

[93] Aragon-Ching J.B., Dahut W.L. (2009). VEGF inhibitors and prostate cancer therapy. Curr. Mol. Pharmacol..

[94] Wong S.Y., Haack H., Crowley D., Barry M., Bronson R.T., Hynes R.O. (2005). Tumor-secreted vascular endothelial growth factor-C is necessary for prostate cancer lymphangiogenesis, but lymphangiogenesis is unnecessary for lymph node metastasis. Cancer Res..

[95] Zhang F., Lee J., Lu S., Pettaway C.A., Dong Z. (2005). Blockade of transforming growth factor-beta signaling suppresses progression of androgen-independent human prostate cancer in nude mice. Clin. Cancer Res..

[96] Tuxhorn J.A., McAlhany S.J., Yang F., Dang T.D., Rowley D.R. (2002). Inhibition of transforming growth factor-beta activity decreases angiogenesis in a human prostate cancer-reactive stroma xenograft model. Cancer Res..

[97] Peinado H., Zhang H., Matei I.R., Costa-Silva B., Hoshino A., Rodrigues G., Psaila B., Kaplan R.N., Bromberg J.F., Kang Y. (2017). Pre-metastatic niches: Organ-specific homes for metastases. Nat. Rev. Cancer.

[98] Arya M., Bott S.R., Shergill I.S., Ahmed H.U., Williamson M., Patel H.R. (2006). The metastatic cascade in prostate cancer. Surg. Oncol..

[99] Lehr J.E., Pienta K.J. (1998). Preferential adhesion of prostate cancer cells to a human bone marrow endothelial cell line. J. Natl. Cancer Inst..

[100] Chin A.R., Wang S.E. (2016). Cancer Tills the Premetastatic Field: Mechanistic Basis and Clinical Implications. Clin. Cancer Res..

[101] Yoneda T., Hiraga T. (2005). Crosstalk between cancer cells and bone microenvironment in bone metastasis. Biochem. Biophys. Res. Commun..

[102] Obenauf A.C., Massagué J. (2015). Surviving at a Distance: Organ-Specific Metastasis. Trends Cancer.

[103] Ren G., Esposito M., Kang Y. (2015). Bone metastasis and the metastatic niche. J. Mol. Med. (Berl.).

[104] Lambert A.W., Pattabiraman D.R., Weinberg R.A. (2017). Emerging Biological Principles of Metastasis. Cell.

[105] Valastyan S., Weinberg R.A. (2011). Tumor metastasis: Molecular insights and evolving paradigms. Cell.

[106] Leber M.F., Efferth T. (2009). Molecular principles of cancer invasion and metastasis (review). Int. J. Oncol..

[107] Kaplan R.N., Riba R.D., Zacharoulis S., Bramley A.H., Vincent L., Costa C., MacDonald D.D., Jin D.K., Shido K., Kerns S.A. (2005). VEGFR1-positive haematopoietic bone marrow progenitors initiate the pre-metastatic niche. Nature.

[108] Taichman R.S., Cooper C., Keller E.T., Pienta K.J., Taichman N.S., McCauley L.K. (2002). Use of the stromal cell-derived factor-1/CXCR4 pathway in prostate cancer metastasis to bone. Cancer Res..

[109] Sun Y.X., Wang J., Shelburne C.E., Lopatin D.E., Chinnaiyan A.M., Rubin M.A., Pienta K.J., Taichman R.S. (2003). Expression of CXCR4 and CXCL12 (SDF-1) in human prostate cancers (PCa) in vivo. J. Cell Biochem..

[110] Shiozawa Y., Pedersen E.A., Havens A.M., Jung Y., Mishra A., Joseph J., Kim J.K., Patel L.R., Ying C., Ziegler A.M. (2011). Human prostate cancer metastases target the hematopoietic stem cell niche to establish footholds in mouse bone marrow. J. Clin. Investig..

[111] Jung Y., Wang J., Lee E., McGee S., Berry J.E., Yumoto K., Dai J., Keller E.T., Shiozawa Y., Taichman R.S. (2015). Annexin 2-CXCL12 interactions regulate metastatic cell targeting and growth in the bone marrow. Mol. Cancer Res..

[112] Diao X., Feng J., Wang Q., Sun J., Chen Z. (2016). SDF-1/CXCR4 axis promotes prostate cancer cell invasion and bone metastasis through p38, NF-kappa B and HIF-1 alpha pathways. Int. J. Clin. Exp. Pathol..

[113] Sottnik J.L., Keller E.T. (2013). Understanding and targeting osteoclastic activity in prostate cancer bone metastases. Curr. Mol. Med..

[114] Yin J.J., Pollock C.B., Kelly K. (2005). Mechanisms of cancer metastasis to the bone. Cell Res..

[115] Ibrahim T., Flamini E., Mercatali L., Sacanna E., Serra P., Amadori D. (2010). Pathogenesis of osteoblastic bone metastases from prostate cancer. Cancer.

[116] Rucci N., Angelucci A. (2014). Prostate Cancer and Bone: The Elective Affinities. Biomed. Res. Int..

[117] Festuccia C., Bologna M., Gravina G.L., Guerra F., Angelucci A., Villanova I., Millimaggi D., Teti A. (1999). Osteoblast conditioned media contain TGF-beta1 and modulate the migration of prostate tumor cells and their interactions with extracellular matrix components. Int. J. Cancer.

[118] Mori K., Le Goff B., Charrier C., Battaglia S., Heymann D., Redini F. (2007). DU145 human prostate cancer cells express functional receptor activator of NF kappa B: New insights in the prostate cancer bone metastasis process. Bone.

[119] Zhang J., Dai J., Qi Y., Lin D.L., Smith P., Strayhorn C., Mizokami A., Fu Z., Westman J., Keller E.T. (2001). Osteoprotegerin inhibits prostate cancer-induced osteoclastogenesis and prevents prostate tumor growth in the bone. J. Clin. Investig..

[120] Pu H., Collazo J., Jones E., Gayheart D., Sakamoto S., Vogt A., Mitchell B., Kyprianou N. (2009). Dysfunctional transforming growth factor-beta receptor II accelerates prostate tumorigenesis in the TRAMP mouse model. Cancer Res..

[121] Cao Z., Kyprianou N. (2015). Mechanisms navigating the TGF-β pathway in prostate cancer. Asian J. Urol..

[122] Ha B., Ko H., Kim B., Sohn E.J., Jung J.H., Kim J.S., Yoon J.J., Won G., Kim J.H., Jung D.B. (2014). Regulation of crosstalk between epithelial to mesenchymal transition molecules and MMP-9 mediates the antimetastatic activity of anethole in DU145 prostate cancer cells. J. Nat. Prod..

[123] Gaetano L., Lorena I., Giuseppe B., Nicola G., Carla F., Marilena C., Giovambattista R., MTF (2006). Activin A circulating levels in patients with bone metastasis from breast or prostate cancer. Clin. Exp. Metastasis.

[124] Hu Z., Gupta J., Zhang Z., Gerseny H., Berg A., Chen Y., Zhang Z., Du H., Brendler C., Xiao X. (2012). Systemic Delivery of Oncolytic Adenoviruses Targeting Transforming Growth Factor-beta Inhibits Established Bone Metastasis in a Prostate Cancer Mouse Model. Hum. Gene Ther..

[125] Wu C.T., Hsieh C.C., Lin C.C., Chen W.C., Hong J.H., Chen M.F. (2012). Significance of IL-6 in the transition of hormone-resistant prostate cancer and the induction of myeloid-derived suppressor cells. J. Mol. Med. (Berl.).

[126] Gu L., Talati P., Vogiatzi P., Romero-Weaver A.L., Abdulghani J., Liao Z., Leiby B., Hoang D.T., Mirtti T., Alanen K. (2014). Pharmacologic suppression of JAK1/2 by JAK1/2 inhibitor AZD1480 potently inhibits IL-6-induced experimental prostate cancer metastases formation. Mol. Cancer Ther.

[127] Kim S.W., Kim J.S., Papadopoulos J., Choi H.J., He J., Maya M., Langley R.R., Fan D., Fidler I.J., Kim S.J. (2011). Consistent interactions between tumor cell IL-6 and macrophage TNF-α enhance the growth of human prostate cancer cells in the bone of nude mouse. Int. Immunopharmacol..

[128] Bellido T., Borba V.Z., Roberson P., Manolagas S.C. (1997). Activation of the Janus kinase/STAT (signal transducer and activator of transcription) signal transduction pathway by interleukin-6-type cytokines promotes osteoblast differentiation. Endocrinology.

[129] Mizutani K., Sud S., Pienta K.J. (2009). Prostate cancer promotes CD11b positive cells to differentiate into osteoclasts. J. Cell Biochem..

[130] Lu Y., Chen Q., Corey E., Xie W., Fan J., Mizokami A., Zhang J. (2009). Activation of MCP-1/CCR2 axis promotes prostate cancer growth in bone. Clin. Exp. Metastasis.

[131] Loberg R.D., Day L.L., Harwood J., Ying C., St John L.N., Giles R., Neeley C.K., Pienta K.J. (2006). CCL2 is a potent regulator of prostate cancer cell migration and proliferation. Neoplasia.

[132] Loberg R.D., Ying C., Craig M., Day L.L., Sargent E., Neeley C., Wojno K., Snyder L.A., Yan L., Pienta K.J. (2007). Targeting CCL2 with systemic delivery of neutralizing antibodies induces prostate cancer tumor regression in vivo. Cancer Res..

[133] Li X., Loberg R., Liao J., Ying C., Snyder L.A., Pienta K.J., McCauley L.K. (2009). A destructive cascade mediated by CCL2 facilitates prostate cancer growth in bone. Cancer Res..

[134] Mizutani K., Sud S., McGregor N.A., Martinovski G., Rice B.T., Craig M.J., Varsos Z.S., Roca H., Pienta K.J. (2009). The chemokine CCL2 increases prostate tumor growth and bone metastasis through macrophage and osteoclast recruitment. Neoplasia.

[135] Lin T.H., Liu H.H., Tsai T.H., Chen C.C., Hsieh T.F., Lee S.S., Lee Y.J., Chen W.C., Tang C.H. (2013). CCL2 increases αvβ3 integrin expression and subsequently promotes prostate cancer migration. Biochim. Biophys. Acta.

[136] Domanska U.M., Kruizinga R.C., Nagengast W.B., Timmer-Bosscha H., Huls G., de Vries E.G., Walenkamp A.M. (2013). A review on CXCR4/CXCL12 axis in oncology: No place to hide. Eur J. Cancer.

[137] Darash-Yahana M., Pikarsky E., Abramovitch R., Zeira E., Pal B., Karplus R., Beider K., Avniel S., Kasem S., Galun E. (2004). Role of high expression levels of CXCR4 in tumor growth, vascularization, and metastasis. FASEB J..

[138] Porvasnik S., Sakamoto N., Kusmartsev S., Eruslanov E., Kim W.J., Cao W., Urbanek C., Wong D., Goodison S., Rosser C.J. (2009). Effects of CXCR4 antagonist CTCE-9908 on prostate tumor growth. Prostate.

[139] Gupta N., Duda D.G. (2016). Role of stromal cell-derived factor 1α pathway in bone metastatic prostate cancer. J. Biomed. Res..

[140] Sun Y.X., Schneider A., Jung Y., Wang J., Dai J., Cook K., Osman N.I., Koh-Paige A.J., Shim H., Pienta K.J. (2005). Skeletal localization and neutralization of the SDF-1(CXCL12)/CXCR4 axis blocks prostate cancer metastasis and growth in osseous sites in vivo. J. Bone Miner. Res..

[141] Wang Q., Diao X., Sun J., Chen Z. (2011). Regulation of VEGF, MMP-9 and metastasis by CXCR4 in a prostate cancer cell line. Cell Biol. Int..

[142] Sun Y.X., Fang M., Wang J., Cooper C.R., Pienta K.J., Taichman R.S. (2007). Expression and activation of alpha v beta 3 integrins by SDF-1/CXC12 increases the aggressiveness of prostate cancer cells. Prostate.

[143] Engl T., Relja B., Marian D., Blumenberg C., Müller I., Beecken W.D., Jones J., Ringel E.M., Bereiter-Hahn J., Jonas D. (2006). CXCR4 chemokine receptor mediates prostate tumor cell adhesion through alpha5 and beta3 integrins. Neoplasia.

[144] Sottnik J.L., Daignault-Newton S., Zhang X., Morrissey C., Hussain M.H., Keller E.T., Hall C.L. (2013). Integrin alpha2beta 1 (α2β1) promotes prostate cancer skeletal metastasis. Clin. Exp. Metastasis.

[145] Hall C.L., Dai J., van Golen K.L., Keller E.T., Long M.W. (2006). Type I collagen receptor (alpha 2 beta 1) signaling promotes the growth of human prostate cancer cells within the bone. Cancer Res..

[146] Odero-Marah V.A., Wang R., Chu G., Zayzafoon M., Xu J., Shi C., Marshall F.F., Zhau H.E., Chung L.W. (2008). Receptor activator of NF-kappaB Ligand (RANKL) expression is associated with epithelial to mesenchymal transition in human prostate cancer cells. Cell Res..

[147] Chu G.C., Zhau H.E., Wang R., Rogatko A., Feng X., Zayzafoon M., Liu Y., Farach-Carson M.C., You S., Kim J. (2014). RANK- and c-Met-mediated signal network promotes prostate cancer metastatic colonization. Endocr. Relat. Cancer.

[148] Armstrong A.P., Miller R.E., Jones J.C., Zhang J., Keller E.T., Dougall W.C. (2008). RANKL acts directly on RANK-expressing prostate tumor cells and mediates migration and expression of tumor metastasis genes. Prostate.

[149] Chen G., Sircar K., Aprikian A., Potti A., Goltzman D., Rabbani S. (2006). Expression of RANKL/RANK/OPG in primary and metastatic human prostate cancer as markers of disease stage and functional regulation. Cancer.

[150] Christoph F., König F., Lebentrau S., Jandrig B., Krause H., Strenziok R., Schostak M. (2018). RANKL/RANK/OPG cytokine receptor system: mRNA expression pattern in BPH, primary and metastatic prostate cancer disease. World J. Urol..

[151] Luo J.L., Tan W., Ricono J.M., Korchynskyi O., Zhang M., Gonias S.L., Cheresh D.A., Karin M. (2007). Nuclear cytokine-activated IKKalpha controls prostate cancer metastasis by repressing Maspin. Nature.

[152] Morrissey C., Kostenuik P., Brown L., Vessella R., Corey E. (2007). Host-derived RANKL is responsible for osteolysis in a C4-2 human prostate cancer xenograft model of experimental bone metastases. BMC Cancer.

[153] Casimiro S., Mohammad K., Pires R., Tato-Costa J., Alho I., Teixeira R., Carvalho A., Ribeiro S., Lipton A., Guise T. (2013). RANKL/RANK/MMP-1 Molecular Triad Contributes to the Metastatic Phenotype of Breast and Prostate Cancer Cells In Vitro. PLoS ONE.

[154] Li Y., He Y., Butler W., Xu L., Chang Y., Lei K., Zhang H., Zhou Y., Gao A.C., Zhang Q. (2019). Targeting cellular heterogeneity with CXCR2 blockade for the treatment of therapy-resistant prostate cancer. Sci. Transl. Med..

[155] Kim S.J., Uehara H., Karashima T., Mccarty M., Shih N., Fidler I.J. (2001). Expression of interleukin-8 correlates with angiogenesis, tumorigenicity, and metastasis of human prostate cancer cells implanted orthotopically in nude mice. Neoplasia.

[156] Inoue K., Slaton J.W., Eve B.Y., Kim S.J., Perrotte P., Balbay M.D., Yano S., Bar-Eli M., Radinsky R., Pettaway C.A. (2000). Interleukin 8 expression regulates tumorigenicity and metastases in androgen-independent prostate cancer. Clin. Cancer Res..

[157] Petreaca M.L., Yao M., Liu Y., Defea K., Martins-Green M. (2007). Transactivation of vascular endothelial growth factor receptor-2 by interleukin-8 (IL-8/CXCL8) is required for IL-8/CXCL8-induced endothelial permeability. Mol. Biol. Cell.

[158] Montecinos V.P., Godoy A., Hinklin J., Vethanayagam R.R., Smith G.J. (2012). Primary xenografts of human prostate tissue as a model to study angiogenesis induced by reactive stroma. PLoS ONE.

[159] Sweeney P., Karashima T., Kim S.J., Kedar D., Mian B., Huang S., Baker C., Fan Z., Hicklin D.J., Pettaway C.A. (2002). Anti-vascular endothelial growth factor receptor 2 antibody reduces tumorigenicity and metastasis in orthotopic prostate cancer xenografts via induction of endothelial cell apoptosis and reduction of endothelial cell matrix metalloproteinase type 9 production. Clin. Cancer Res..

[160] Dai J., Kitagawa Y., Zhang J., Yao Z., Mizokami A., Cheng S., Nör J., McCauley L.K., Taichman R.S., Keller E.T. (2004). Vascular endothelial growth factor contributes to the prostate cancer-induced osteoblast differentiation mediated by bone morphogenetic protein. Cancer Res..

[161] Kitagawa Y., Dai J., Zhang J., Keller J.M., Nor J., Yao Z., Keller E.T. (2005). Vascular endothelial growth factor contributes to prostate cancer-mediated osteoblastic activity. Cancer Res..

[162] De S., Chen J., Narizhneva N.V., Heston W., Brainard J., Sage E.H., Byzova T.V. (2003). Molecular pathway for cancer metastasis to bone. J. Biol. Chem..

[163] Lee Y., Schwarz E., Davies M., Jo M., Gates J., Wu J., Zhang X., Lieberman J.R. (2003). Differences in the cytokine profiles associated with prostate cancer cell induced osteoblastic and osteolytic lesions in bone. J. Orthop. Res..

[164] Kuo P.L., Shen K.H., Hung S.H., Hsu Y.L. (2012). CXCL1/GROα increases cell migration and invasion of prostate cancer by decreasing fibulin-1 expression through NF-κB/HDAC1 epigenetic regulation. Carcinogenesis.

[165] Lu Y., Dong B., Xu F., Xu Y., Pan J., Song J., Zhang J., Huang Y., Xue W. (2019). CXCL1-LCN2 paracrine axis promotes progression of prostate cancer via the Src activation and epithelial-mesenchymal transition. Cell Commun. Signal..

[166] Jung Y., Kim J.K., Shiozawa Y., Wang J., Mishra A., Joseph J., Berry J.E., McGee S., Lee E., Sun H. (2013). Recruitment of mesenchymal stem cells into prostate tumours promotes metastasis. Nat. Commun..

[167] Hu W., Zhen X., Xiong B., Wang B., Zhang W., Zhou W. (2008). CXCR6 is expressed in human prostate cancer in vivo and is involved in the in vitro invasion of PC3 and LNCap cells. Cancer Sci..

[168] Singh R., Kapur N., Mir H., Singh N., Lillard J.W., Singh S. (2016). CXCR6-CXCL16 axis promotes prostate cancer by mediating cytoskeleton rearrangement via Ezrin activation and αvβ3 integrin clustering. Oncotarget.

[169] Lebrun J.J. (2012). The Dual Role of TGFβ in Human Cancer: From Tumor Suppression to Cancer Metastasis. ISRN Mol. Biol..

[170] Padua D., Massagué J. (2009). Roles of TGFbeta in metastasis. Cell Res..

[171] Jones E., Pu H., Kyprianou N. (2009). Targeting TGF-beta in prostate cancer: Therapeutic possibilities during tumor progression. Expert Opin. Ther. Targets.

[172] Bellomo C., Caja L., Moustakas A. (2016). Transforming growth factor β as regulator of cancer stemness and metastasis. Br. J. Cancer.

[173] Xie F., Ling L., van Dam H., Zhou F., Zhang L. (2018). TGF-β signaling in cancer metastasis. Acta Biochim. Biophys. Sin. (Shanghai).

[174] Hao Y., Baker D., Ten Dijke P. (2019). TGF-β-Mediated Epithelial-Mesenchymal Transition and Cancer Metastasis. Int. J. Mol. Sci..

[175] Ahel J., Hudorović N., Vičić-Hudorović V., Nikles H. (2019). TGF-BETA In The Natural History Of Prostate Cancer. Acta Clin. Croat..

[176] Wikström P., Stattin P., Franck-Lissbrant I., Damber J.E., Bergh A. (1998). Transforming growth factor beta1 is associated with angiogenesis, metastasis, and poor clinical outcome in prostate cancer. Prostate.

[177] Tsubakihara Y., Moustakas A. (2018). Epithelial-Mesenchymal Transition and Metastasis under the Control of Transforming Growth Factor β. Int. J. Mol. Sci..

[178] Hansen A.G., Arnold S.A., Jiang M., Palmer T.D., Ketova T., Merkel A., Pickup M., Samaras S., Shyr Y., Moses H.L. (2014). ALCAM/CD166 is a TGF-β-responsive marker and functional regulator of prostate cancer metastasis to bone. Cancer Res..

[179] Tu W.H., Thomas T.Z., Masumori N., Bhowmick N.A., Gorska A.E., Shyr Y., Kasper S., Case T., Roberts R.L., Shappell S.B. (2003). The loss of TGF-beta signaling promotes prostate cancer metastasis. Neoplasia.

[180] Ding Z., Wu C.J., Chu G.C., Xiao Y., Ho D., Zhang J., Perry S.R., Labrot E.S., Wu X., Lis R. (2011). SMAD4-dependent barrier constrains prostate cancer growth and metastatic progression. Nature.

[181] Mishra S., Tang Y., Wang L., deGraffenried L., Yeh I.T., Werner S., Troyer D., Copland J.A., Sun L.Z. (2011). Blockade of transforming growth factor-beta (TGFβ) signaling inhibits osteoblastic tumorigenesis by a novel human prostate cancer cell line. Prostate.

[182] Culig Z. (2014). Proinflammatory cytokine interleukin-6 in prostate carcinogenesis. Am. J. Clin. Exp. Urol..

[183] Nguyen D.P., Li J., Tewari A.K. (2014). Inflammation and prostate cancer: The role of interleukin 6 (IL-6). BJU Int..

[184] Ara T., Declerck Y.A. (2010). Interleukin-6 in bone metastasis and cancer progression. Eur J. Cancer.

[185] Nakashima J., Tachibana M., Horiguchi Y., Oya M., Ohigashi T., Asakura H., Murai M. (2000). Serum interleukin 6 as a prognostic factor in patients with prostate cancer. Clin. Cancer Res..

[186] Michalaki V., Syrigos K., Charles P., Waxman J. (2004). Serum levels of IL-6 and TNF-alpha correlate with clinicopathological features and patient survival in patients with prostate cancer. Br. J. Cancer.

[187] George D.J., Halabi S., Shepard T.F., Sanford B., Vogelzang N.J., Small E.J., Kantoff P.W. (2005). The prognostic significance of plasma interleukin-6 levels in patients with metastatic hormone-refractory prostate cancer: Results from cancer and leukemia group B 9480. Clin. Cancer Res..

[188] Shariat S.F., Andrews B., Kattan M.W., Kim J., Wheeler T.M., Slawin K.M. (2001). Plasma levels of interleukin-6 and its soluble receptor are associated with prostate cancer progression and metastasis. Urology.

[189] Kuroda K., Nakashima J., Kanao K., Kikuchi E., Miyajima A., Horiguchi Y., Nakagawa K., Oya M., Ohigashi T., Murai M. (2007). Interleukin 6 is associated with cachexia in patients with prostate cancer. Urology.

[190] Santer F.R., Malinowska K., Culig Z., Cavarretta I.T. (2010). Interleukin-6 trans-signalling differentially regulates proliferation, migration, adhesion and maspin expression in human prostate cancer cells. Endocr. Relat. Cancer.

[191] Morrissey C., Lai J.S., Brown L.G., Wang Y.C., Roudier M.P., Coleman I.M., Gulati R., Vakar-Lopez F., True L.D., Corey E. (2010). The expression of osteoclastogenesis-associated factors and osteoblast response to osteolytic prostate cancer cells. Prostate.

[192] Pencik J., Schlederer M., Gruber W., Unger C., Walker S.M., Chalaris A., Marié I.J., Hassler M.R., Javaheri T., Aksoy O. (2015). STAT3 regulated ARF expression suppresses prostate cancer metastasis. Nat. Commun..

[193] Wang X., Lee S.O., Xia S., Jiang Q., Luo J., Li L., Yeh S., Chang C. (2013). Endothelial cells enhance prostate cancer metastasis via IL-6→androgen receptor→TGF-β→MMP-9 signals. Mol. Cancer Ther..

[194] Lim S.Y., Yuzhalin A.E., Gordon-Weeks A.N., Muschel R.J. (2016). Targeting the CCL2-CCR2 signaling axis in cancer metastasis. Oncotarget.

[195] Zhang J., Lu Y., Pienta K.J. (2010). Multiple roles of chemokine (C-C motif) ligand 2 in promoting prostate cancer growth. J. Natl. Cancer Inst..

[196] Lu Y., Cai Z., Galson D.L., Xiao G., Liu Y., George D.E., Melhem M.F., Yao Z., Zhang J. (2006). Monocyte chemotactic protein-1 (MCP-1) acts as a paracrine and autocrine factor for prostate cancer growth and invasion. Prostate.

[197] Izhak L., Wildbaum G., Weinberg U., Uri W., Shaked Y., Alami J., Dumont D., Friedman B., Stein A., Karin N. (2010). Predominant expression of CCL2 at the tumor site of prostate cancer patients directs a selective loss of immunological tolerance to CCL2 that could be amplified in a beneficial manner. J. Immunol..

[198] Lu Y., Xiao G., Galson D.L., Nishio Y., Mizokami A., Keller E.T., Yao Z., Zhang J. (2007). PTHrP-induced MCP-1 production by human bone marrow endothelial cells and osteoblasts promotes osteoclast differentiation and prostate cancer cell proliferation and invasion in vitro. Int. J. Cancer.

[199] Sun X., Cheng G., Hao M., Zheng J., Zhou X., Zhang J., Taichman R., Pienta K., Wang J. (2010). CXCL12/CXCR4/CXCR7 chemokine axis and cancer progression. Cancer Metastasis Rev..

[200] Mochizuki H., Matsubara A., Teishima J., Mutaguchi K., Yasumoto H., Dahiya R., Usui T., Kamiya K. (2004). Interaction of ligand-receptor system between stromal-cell-derived factor-1 and CXC chemokine receptor 4 in human prostate cancer: A possible predictor of metastasis. Biochem. Biophys. Res. Commun..

[201] Xing Y., Liu M., Du Y., Qu F., Li Y., Zhang Q., Xiao Y., Zhao J., Zeng F., Xiao C. (2008). Tumor cell-specific blockade of CXCR4/SDF-1 interactions in prostate cancer cells by hTERT promoter induced CXCR4 knockdown: A possible metastasis preventing and minimizing approach. Cancer Biol. Ther..

[202] Gladson C.L., Welch D.R. (2008). New insights into the role of CXCR4 in prostate cancer metastasis. Cancer Biol. Ther..

[203] Singh S., Singh U.P., Grizzle W.E., Lillard J.W. (2004). CXCL12-CXCR4 interactions modulate prostate cancer cell migration, metalloproteinase expression and invasion. Lab. Investig..

[204] Chinni S.R., Sivalogan S., Dong Z., Filho J.C., Deng X., Bonfil R.D., Cher M.L. (2006). CXCL12/CXCR4 signaling activates Akt-1 and MMP-9 expression in prostate cancer cells: The role of bone microenvironment-associated CXCL12. Prostate.

[205] Wang Q., Diao X., Sun J., Chen Z. (2013). Stromal cell-derived factor-1 and vascular endothelial growth factor as biomarkers for lymph node metastasis and poor cancer-specific survival in prostate cancer patients after radical prostatectomy. Urol. Oncol..

[206] Wang J., Dai J., Jung Y., Wei C.L., Wang Y., Havens A.M., Hogg P.J., Keller E.T., Pienta K.J., Nor J.E. (2007). A glycolytic mechanism regulating an angiogenic switch in prostate cancer. Cancer Res..

[207] Jung Y., Cackowski F.C., Yumoto K., Decker A.M., Wang J., Kim J.K., Lee E., Wang Y., Chung J.S., Gursky A.M. (2018). CXCL12γ Promotes Metastatic Castration-Resistant Prostate Cancer by Inducing Cancer Stem Cell and Neuroendocrine Phenotypes. Cancer Res..

[208] Dubrovska A., Elliott J., Salamone R.J., Telegeev G.D., Stakhovsky A.E., Schepotin I.B., Yan F., Wang Y., Bouchez L.C., Kularatne S.A. (2012). CXCR4 expression in prostate cancer progenitor cells. PLoS ONE.

[209] Wang J., Shiozawa Y., Wang Y., Jung Y., Pienta K.J., Mehra R., Loberg R., Taichman R.S. (2008). The role of CXCR7/RDC1 as a chemokine receptor for CXCL12/SDF-1 in prostate cancer. J. Biol. Chem..

[210] Singh R.K., Lokeshwar B.L. (2011). The IL-8-regulated chemokine receptor CXCR7 stimulates EGFR signaling to promote prostate cancer growth. Cancer Res..

[211] Li S., Fong K.W., Gritsina G., Zhang A., Zhao J.C., Kim J., Sharp A., Yuan W., Aversa C., Yang X.J. (2019). Activation of MAPK Signaling by CXCR7 Leads to Enzalutamide Resistance in Prostate Cancer. Cancer Res..

[212] Saha A., Ahn S., Blando J., Su F., Kolonin M.G., DiGiovanni J. (2017). Proinflammatory CXCL12-CXCR4/CXCR7 Signaling Axis Drives Myc-Induced Prostate Cancer in Obese Mice. Cancer Res..

[213] Luo Y., Azad A.K., Karanika S., Basourakos S.P., Zuo X., Wang J., Yang L., Yang G., Korentzelos D., Yin J. (2018). Enzalutamide and CXCR7 inhibitor combination treatment suppresses cell growth and angiogenic signaling in castration-resistant prostate cancer models. Int. J. Cancer.

[214] Boyce B., Xing L. (2007). Biology of RANK, RANKL, and osteoprotegerin. Arthritis Res. Ther..

[215] Sisay M., Mengistu G., Edessa D. (2017). The RANK/RANKL/OPG system in tumorigenesis and metastasis of cancer stem cell: Potential targets for anticancer therapy. Oncotargets Ther..

[216] Teitelbaum S.L., Ross F.P. (2003). Genetic regulation of osteoclast development and function. Nat. Rev. Genet..

[217] Ikeda T., Kasai M., Utsuyama M., Hirokawa K. (2001). Determination of three isoforms of the receptor activator of nuclear factor-kappa B ligand and their differential expression in bone and thymus. Endocrinology.

[218] Penno H., Nilsson O., Brandstrom H., Winqvist O., Ljunggren O. (2009). Expression of RANK-ligand in prostate cancer cell lines. Scand. J. Clin. Lab. Investig..

[219] Miller R., Roudier M., Jones J., Armstrong A., Canon J., Dougall W. (2008). RANK ligand inhibition plus docetaxel improves survival and reduces tumor burden in a murine model of prostate cancer bone metastasis. Mol. Cancer Ther..

[220] Takayama K., Inoue T., Narita S., Maita S., Huang M., Numakura K., Tsuruta H., Saito M., Maeno A., Satoh S. (2017). Inhibition of the RANK/RANKL signaling with osteoprotegerin prevents castration-induced acceleration of bone metastasis in castration-insensitive prostate cancer. Cancer Lett..

[221] Maxwell P.J., Gallagher R., Seaton A., Wilson C., Scullin P., Pettigrew J., Stratford I.J., Williams K.J., Johnston P.G., Waugh D.J. (2007). HIF-1 and NF-kappaB-mediated upregulation of CXCR1 and CXCR2 expression promotes cell survival in hypoxic prostate cancer cells. Oncogene.

[222] Guo Y., Zang Y., Lv L., Cai F., Qian T., Zhang G., Feng Q. (2017). IL-8 promotes proliferation and inhibition of apoptosis via STAT3/AKT/NF-κB pathway in prostate cancer. Mol. Med. Rep..

[223] Lehrer S., Diamond E.J., Mamkine B., Stone N.N., Stock R.G. (2004). Serum interleukin-8 is elevated in men with prostate cancer and bone metastases. Technol. Cancer Res. Treat..

[224] Murphy C., McGurk M., Pettigrew J., Santinelli A., Mazzucchelli R., Johnston P.G., Montironi R., Waugh D.J. (2005). Nonapical and cytoplasmic expression of interleukin-8, CXCR1, and CXCR2 correlates with cell proliferation and microvessel density in prostate cancer. Clin. Cancer Res..

[225] Araki S., Omori Y., Lyn D., Singh R.K., Meinbach D.M., Sandman Y., Lokeshwar V.B., Lokeshwar B.L. (2007). Interleukin-8 is a molecular determinant of androgen independence and progression in prostate cancer. Cancer Res..

[226] Maynard J.P., Ertunc O., Kulac I., Baena-Del Valle J.A., De Marzo A.M., Sfanos K.S. (2020). IL8 Expression Is Associated with Prostate Cancer Aggressiveness and Androgen Receptor Loss in Primary and Metastatic Prostate Cancer. Mol. Cancer Res..

[227] Terry S., Beltran H. (2014). The many faces of neuroendocrine differentiation in prostate cancer progression. Front. Oncol..

[228] Lu Y., Cai Z., Xiao G., Keller E.T., Mizokami A., Yao Z., Roodman G.D., Zhang J. (2007). Monocyte chemotactic protein-1 mediates prostate cancer-induced bone resorption. Cancer Res..

[229] Liu J.F., Tsao Y.T., Hou C.H. (2017). Fractalkine/CX3CL1 induced intercellular adhesion molecule-1-dependent tumor metastasis through the CX3CR1/PI3K/Akt/NF-κB pathway in human osteosarcoma. Oncotarget.

[230] Ferretti E., Pistoia V., Corcione A. (2014). Role of fractalkine/CX3CL1 and its receptor in the pathogenesis of inflammatory and malignant diseases with emphasis on B cell malignancies. Mediat. Inflamm..

[231] Marchesi F., Piemonti L., Mantovani A., Allavena P. (2010). Molecular mechanisms of perineural invasion, a forgotten pathway of dissemination and metastasis. Cytokine Growth Factor Rev..

[232] Liu P., Liang Y., Jiang L., Wang H., Wang S., Dong J. (2018). CX3CL1/fractalkine enhances prostate cancer spinal metastasis by activating the Src/FAK pathway. Int. J. Oncol..

[233] Shulby S.A., Dolloff N.G., Stearns M.E., Meucci O., Fatatis A. (2004). CX3CR1-fractalkine expression regulates cellular mechanisms involved in adhesion, migration, and survival of human prostate cancer cells. Cancer Res..

[234] Jamieson W.L., Shimizu S., D’Ambrosio J.A., Meucci O., Fatatis A. (2008). CX3CR1 is expressed by prostate epithelial cells and androgens regulate the levels of CX3CL1/fractalkine in the bone marrow: Potential role in prostate cancer bone tropism. Cancer Res..

[235] Ferrer F.A., Miller L.J., Andrawis R.I., Kurtzman S.H., Albertsen P.C., Laudone V.P., Kreutzer D.L. (1997). Vascular endothelial growth factor (VEGF) expression in human prostate cancer: In situ and in vitro expression of VEGF by human prostate cancer cells. J. Urol..

[236] Green M.M., Hiley C.T., Shanks J.H., Bottomley I.C., West C.M., Cowan R.A., Stratford I.J. (2007). Expression of vascular endothelial growth factor (VEGF) in locally invasive prostate cancer is prognostic for radiotherapy outcome. Int. J. Radiat. Oncol. Biol. Phys..

[237] Chen J., De S., Brainard J., Byzova T.V. (2004). Metastatic properties of prostate cancer cells are controlled by VEGF. Cell Commun. Adhes.

[238] Botelho F., Pina F., Lunet N. (2010). VEGF and prostatic cancer: A systematic review. Eur. J. Cancer Prev..

[239] Duque J.L., Loughlin K.R., Adam R.M., Kantoff P.W., Zurakowski D., Freeman M.R. (1999). Plasma levels of vascular endothelial growth factor are increased in patients with metastatic prostate cancer. Urology.

[240] Jackson M.W., Roberts J.S., Heckford S.E., Ricciardelli C., Stahl J., Choong C., Horsfall D.J., Tilley W.D. (2002). A potential autocrine role for vascular endothelial growth factor in prostate cancer. Cancer Res..

[241] Yang L., You S., Kumar V., Zhang C., Cao Y. (2012). In vitro the behaviors of metastasis with suppression of VEGF in human bone metastatic LNCaP-derivative C4-2B prostate cancer cell line. J. Exp. Clin. Cancer Res..

[242] de Brot S., Ntekim A., Cardenas R., James V., Allegrucci C., Heery D.M., Bates D.O., Ødum N., Persson J.L., Mongan N.P. (2015). Regulation of vascular endothelial growth factor in prostate cancer. Endocr. Relat. Cancer.

[243] Liu Q., Russell M.R., Shahriari K., Jernigan D.L., Lioni M.I., Garcia F.U., Fatatis A. (2013). Interleukin-1β promotes skeletal colonization and progression of metastatic prostate cancer cells with neuroendocrine features. Cancer Res..

[244] Abdul M., Hoosein N. (2000). Differences in the expression and effects of interleukin-1 and -2 on androgen-sensitive and -insensitive human prostate cancer cell lines. Cancer Lett..

[245] Thomas-Jardin S.E., Kanchwala M.S., Jacob J., Merchant S., Meade R.K., Gahnim N.M., Nawas A.F., Xing C., Delk N.A. (2018). Identification of an IL-1-induced gene expression pattern in AR. Prostate.

[246] Miyake M., Lawton A., Goodison S., Urquidi V., Rosser C.J. (2014). Chemokine (C-X-C motif) ligand 1 (CXCL1) protein expression is increased in high-grade prostate cancer. Pathol. Res. Pract..

[247] Hardaway A.L., Herroon M.K., Rajagurubandara E., Podgorski I. (2015). Marrow adipocyte-derived CXCL1 and CXCL2 contribute to osteolysis in metastatic prostate cancer. Clin. Exp. Metastasis.

[248] Schroten C., Dits N.F., Steyerberg E.W., Kranse R., van Leenders A.G., Bangma C.H., Kraaij R. (2012). The additional value of TGFβ1 and IL-7 to predict the course of prostate cancer progression. Cancer Immunol. Immunother..

[249] Qu H., Zou Z., Pan Z., Zhang T., Deng N., Chen G., Wang Z. (2016). IL-7/IL-7 receptor axis stimulates prostate cancer cell invasion and migration via AKT/NF-κB pathway. Int. Immunopharmacol..

[250] Mengus C., Le Magnen C., Trella E., Yousef K., Bubendorf L., Provenzano M., Bachmann A., Heberer M., Spagnoli G.C., Wyler S. (2011). Elevated levels of circulating IL-7 and IL-15 in patients with early stage prostate cancer. J. Transl. Med..

[251] Lu Y., Wang J., Xu Y., Koch A.E., Cai Z., Chen X., Galson D.L., Taichman R.S., Zhang J. (2008). CXCL16 functions as a novel chemotactic factor for prostate cancer cells in vitro. Mol. Cancer Res..

[252] Wang J., Lu Y., Koch A.E., Zhang J., Taichman R.S. (2008). CXCR6 induces prostate cancer progression by the AKT/mammalian target of rapamycin signaling pathway. Cancer Res..

[253] Darash-Yahana M., Gillespie J.W., Hewitt S.M., Chen Y.Y., Maeda S., Stein I., Singh S.P., Bedolla R.B., Peled A., Troyer D.A. (2009). The chemokine CXCL16 and its receptor, CXCR6, as markers and promoters of inflammation-associated cancers. PLoS ONE.

[254] Ha H.K., Lee W., Park H.J., Lee S.D., Lee J.Z., Chung M.K. (2011). Clinical significance of CXCL16/CXCR6 expression in patients with prostate cancer. Mol. Med. Rep..

[255] Richardsen E., Ness N., Melbø-Jørgensen C., Johannesen C., Grindstad T., Nordbakken C., Al-Saad S., Andersen S., Dønnem T., Nordby Y. (2015). The prognostic significance of CXCL16 and its receptor C-X-C chemokine receptor 6 in prostate cancer. Am. J. Pathol..

